# Estimating outflow facility through pressure dependent pathways of the human eye

**DOI:** 10.1371/journal.pone.0188769

**Published:** 2017-12-20

**Authors:** David W. Smith, Bruce S. Gardiner

**Affiliations:** 1 Faculty of Engineering and Mathematical Sciences, The University of Western Australia, Perth, Australia; 2 School of Engineering and Information Technology, Murdoch University, Murdoch, Western Australia, Australia; Oregon Health and Science University, UNITED STATES

## Abstract

We develop and test a new theory for pressure dependent outflow from the eye. The theory comprises three main parameters: (i) a constant hydraulic conductivity, (ii) an exponential decay constant and (iii) a no-flow intraocular pressure, from which the total pressure dependent outflow, average outflow facilities and local outflow facilities for the whole eye may be evaluated. We use a new notation to specify precisely the meaning of model parameters and so model outputs. Drawing on a range of published data, we apply the theory to animal eyes, enucleated eyes and *in vivo* human eyes, and demonstrate how to evaluate model parameters. It is shown that the theory can fit high quality experimental data remarkably well. The new theory predicts that outflow facilities and total pressure dependent outflow for the whole eye are more than twice as large as estimates based on the Goldman equation and fluorometric analysis of anterior aqueous outflow. It appears likely that this discrepancy can be largely explained by pseudofacility and aqueous flow through the retinal pigmented epithelium, while any residual discrepancy may be due to pathological processes in aged eyes. The model predicts that if the hydraulic conductivity is too small, or the exponential decay constant is too large, then intraocular eye pressure may become unstable when subjected to normal circadian changes in aqueous production. The model also predicts relationships between variables that may be helpful when planning future experiments, and the model generates many novel testable hypotheses. With additional research, the analysis described here may find application in the differential diagnosis, prognosis and monitoring of glaucoma.

## Introduction

Glaucoma is the most significant cause of irreversible blindness world-wide, with some 70 million people affected [1]. While glaucoma is a group of diseases, there is a crucially important association between the initiation and progression of glaucoma, and raised intraocular pressure (IOP). It is hypothesized that raised IOP (or more specifically, elevation of the pressure gradient across the optic nerve head [2, 3]) can directly lead to optic nerve neuropathy. One postulated mechanism driving optic nerve neuropathy is the disruption of axonal transport at the optic nerve head, which leads to retinal ganglion cell degeneration and loss of vision [4, 5]. The only proven treatment of glaucoma is reduction of IOP [1, 6].

Given the importance of IOP from diagnostic, monitoring and prognostic viewpoints, trying to measure and understand the causes of elevated intraocular pressure has attracted considerable attention. It is known that IOP is determined by the interplay between net fluid production and pressure dependent outflow from the eye. Outflow occurs through the trabecular meshwork (the so-called ‘conventional pathway’) and the through the uveoscleral route (the so-called ‘unconventional pathway’) [7]. But it is also well-documented that pressure dependent and pressure independent outflow also occurs across the retinal pigmented epithelium into the choroid [8–18]. Probably because it is difficult to measure clinically [19], this latter pathway is often neglected in discussions of eye fluid turnover [6, 20, 21]. Further pressure driven outflow may also occur at the ciliary body itself [22–26], which is the usual source of fluid production in the eye [27, 28].

Impairment of outflow facility of the eye is believed to be the primary determinant of raised IOP [29]. Consequently there is considerable clinical interest in measuring the outflow facility, both to identify a primary risk for glaucoma and to monitor the IOP response to treatment (e.g. drug therapies). Friedenwald (1937) realized that accurate clinical estimation of local outflow facility using tonography depended on an accurate estimation of the ocular rigidity. This led him to test the ocular rigidity of eyes *ex vivo*, and to develop nomograms to interpret paired Schiotz tonometric measurements [30–33]. Indeed, ocular rigidity can be directly measured in a clinical setting using differential tonometry, which may involve use of Schiotz tonometry and pneumatonometer or Goldman tonometry [19, 31–35]. Alternatively more elaborate means may be employed, such as measuring intraocular pressure pulsation in response to pulsatile blood flow [36] or using Pascal dynamic contour tonometry to measure ocular pressure pulsations, while measuring changes in choroidal thickness using optical coherence tomography to estimate blood pulsation volume within the eye [37, 38].

Initially outflow facility of the eye was thought to be constant [39–41], though it was recognised that outflow facility decreased in some disease states [40]. However, beginning with Brubaker (1975) and Moses (1977), it became increasingly apparent that outflow facility is pressure dependent [42–44]. More recently high quality evidence has emerged indicating outflow facility is indeed a function of intraocular pressure for normal eyes. This is clearly evidenced in both animal eyes [45, 46] and *in vivo* human eyes [47, 48]. This pressure dependence of outflow facility sets new challenges in interpreting outflow facility measurements, and for identifying exactly what changes occur in disease states such as glaucoma.

In this paper we develop a new theoretical model for the analysis of pressure dependent outflow from the eye. To unambiguously develop this theory requires much greater precision in the way outflow facility (*C*) has been defined than heretofore [49]. Indeed when outflow facility is pressure dependent, there is any number of outflow facilities that can be measured for an eye. For this reason, we need to introduce a new notation that removes any possible uncertainty or ambiguity about precisely which outflow facility is being considered. Here we define and so distinguish two quantities:

1. a local (or point) estimate of outflow facility, as the mathematical derivative of the total pressure dependent outflow with respect to IOP (denoted $C_{}^{p_{1}}$ at IOP *p*_1_). For example *C*^15^ (where *C*^15^ means the derivative of the total pressure dependent outflow with respect to IOP evaluated at an IOP of 15 mm Hg);
2. an average of local outflow facility over a pressure range (denoted ${\bar{C}}_{p_{1}}^{p_{2}}$ over a pressure range *p*_1_ to *p*_2_). For example ${\bar{C}}_{15}^{20}$ (where the overbar denotes average, and ${\bar{C}}_{15}^{20}$ means the average of the local outflow facilities over the IOPs ranging from 15 mm Hg to 20 mm Hg).

We find the clinically measured outflow facility corresponds to the second definition, and represents an average of local outflow facilities across a pressure range. We develop the theory and governing equations using the new nomenclature, and then employing various published experimental data, solve the model equations to make estimates of the three key parameters governing outflow facility in the new theoretical model, namely: (i) the hydraulic outflow conductance for the whole eye, $C_{T}^{SL}$ (microliters/min/mm Hg), describing membrane outflow properties (ii) an exponential decay constant *α* (mm Hg)^-1^ for the whole eye, which describes the rate of decrease of local outflow and outflow facility with increasing IOP, and (iii), the IOP denoted *p*_*T*_ (mm Hg), at which there is no pressure dependent flow to or from the eye. Following parameter estimation, we can obtain an average outflow facility for the eye up to the IOP of interest, from which the total pressure dependent outflow from the whole eye may be calculated.

When developing the theory, we make use of physiological fundamentals describing fluid flow across biological membranes to motivate the analysis. We carefully develop the theory that enables us to interpret the various outflow facilities from pressure-volume and pressure-time data obtained from intracameral manometric measurements on the eye. During the theoretical development, we arrive at the position where we need to estimate the difference in the rates of change of IOP and ‘membrane reference pressure’ with respect to changing IOP. Though other functions may be employed, we choose to approximate this relationship as an exponentially decaying function of intraocular pressure, and then follow through the consequences of this assumption, calculating both local and average outflow facilities for the eye.

Having developed the theory and governing equations, we solve the equations to make estimates of the three key model parameters governing pressure dependent outflow. To do this we employ published experimental data on manometric measurements for normal aged *in vivo* human eyes, and published experimental data on manometric measurements for enucleated human eyes. During this calibration, we also refer to and make some use of animal data. Taken together, this experimental data allows us to estimate the total pressure dependent outflow and outflow facility for the normal, aged human eye *in vivo*. Based on experimental measurements, we estimate a total pressure dependent outflow for normal, aged *in vivo* human eyes to be more than twice as large as usual estimates of aqueous production. The theory also reveals that as the hydraulic conductance constant decreases and exponential decay constant increases, intraocular pressure may become unstable with normal circadian variations in net fluid production. Finally the theory suggests many new hypotheses that can be tested with additional experimental data. We begin our analysis by developing our theory of pressure dependent outflow.

## Method

### Development of a theory for pressure dependent outflow

It is well known that fluid flow is driven by spatial differences in the chemical potential of the fluid (for water the chemical potential may be denoted *μ*^*w*^), and fluid flow across any membrane is not an exception. In general a biological ‘membrane’ may refer to a cell membrane, or a cell layer, or extracellular matrix, or some combination thereof. An exemplar case in point motivating our analysis is the net flow of water across a semipermeable membrane separating two ideal solutions, which is described by the Starling force equation [50], viz,

$$\dot{V}=L_{p}^{}(\mu_{}^{w}-\mu_{ref}^{w})=L_{p}^{}[(p-\sigma RTc)-(p_{ref}-\sigma RTc_{ref})]$$ (1)

where $\dot{V}$ is the rate of water volume flux per unit area across the membrane, *R* the gas constant, *T* the absolute temperature in degrees Kelvin. $\mu_{ref}^{w}$, *p*_*ref*_ and *c*_*ref*_ are the reference chemical potential, membrane reference fluid pressure and molar reference concentration of osmotically active particles (which may include proteins and salt depending on the composition of the solution and the pore size of the membrane) on the reference side of the membrane. *μ*^*w*^, *p* and *π* are similar quantities on the other side of the membrane. *L*_*p*_ is the hydraulic filtration coefficient per unit (apparent) cross-sectional area normal to the flow and *σ* is the reflection coefficient that varies between 0 and 1, becoming 1 for ideal membranes. In the following we assume the membrane is ideal, but the theory is easily modified to account for non-ideal conditions.

The hydraulic filtration coefficient *L*_*p*_ is a function that depends on the geometry of the membrane and the viscosity of the fluid. Consequently it is in general a function of other variables, including time. For example, the filtration coefficient may change due to changes in material properties of the membrane (e.g. the aquaporin density of a cell membrane may increase over time, which increases the flow capacity of a cell membrane). Because cells have multiple sensors, gene expression may change with mechanical strain, hydrostatic pressure and osmotic pressure [6, 51], and this may change membrane hydraulic conductance. Naturally we can include these relationships in *L*_*p*_ if they are known, but in the following we assume *L*_*p*_ is constant. This is probably a reasonable approximation for short-term tests that do not involve excessive changes in intraocular pressure [51].

An example of flow across a membrane due to Starling forces occurs at the ciliary body. Ion pumping at the ciliary body results in a relative ion excess in the aqueous humor (i.e. it is a hypertonic solution), which generates an osmotic suction at the aqueous humor. This suction drives water movement from the ciliary body to the aqueous humor [24, 27]. We note that the difference in osmolality across a cell layer (e.g. across the non-pigmented epithelium in the ciliary body) does not need to be very large to generate a significant osmotic suction. For example if the osmolarity difference across the semipermeable membrane Δ*c* = 1 mM, then at 37 degrees Celsius, *RT* Δ*c* = Δ*π* is about 20 mm Hg. So comparatively small differences in concentrations of osmotically active particles across a membrane causes comparatively large changes in equilibrium osmotic pressure.

To illustrate this point, at the choroid plexus in the ventricles of the brain, a difference of 5 mOsm creates an osmotic suction of about 100 mm Hg in the cerebrospinal fluid, drawing water across the choroid plexus epithelium [52]. While the magnitude of the osmotic suction for aqueous humor appears to have not been quantified precisely, it is known aqueous humor is not a simple blood filtrate, as chloride ions are concentrated in the aqueous humor relative to blood plasma (by about 20%), as are some other molecules (e.g. lactate is concentrated 2.4 fold, ascorbic acid is concentrated 50 to 70 fold) (see Table 1 [27]).

Though equilibrium is never reached *in vivo*, at thermodynamic equilibrium the mechanical fluid pressure *p* becomes equal to the osmotic pressure (*π*), so it is usual to replace *RTc* by *π*. For transient ‘pressure dependent’ flow in or out of the eye we can write:

$$\dot{V}=L_{p}^{}[(p-\pi )-(p_{ref}-\pi_{ref})]$$ (2)

As biological membranes contain ion pumps, which play a key role in determining the osmotic pressure difference (Δ*π* = *π* − *π*_*ref*_) across the semipermeable membrane, we may prefer to write the previous equation as,

$$\dot{V}=L_{p}^{}[\Delta p-\Delta \pi )]$$ (3)

where Δ*p* = *p* − *p*_*ref*_. Both intraocular mechanical and osmotic pressures are maintained by Na^+^-K^+^-ATPase (and other) ion pumps—water simply follows the ion gradient established by the ion pumps [27] at a rate determined by the basal membrane permeability and aquaporin density [53]. Ion pumps maintain net fluid flow into the eye from the non-pigmented epithelium at the ciliary body, while at the retinal pigmented epithelium ion pumps maintain a constant outflow from the vitreous to the choroid. Fluid flow across the ciliary body continues because fluid drains or is transported away from the anterior, posterior and vitreous chambers of the eye, so that thermodynamic equilibrium for the system is never established.

It is reported that the ion pumps are dependent for their activity on adequate blood flow [28], as presumably their pumping activity can be limited by their oxygen supply. Importantly, it has been shown that physiological intraocular pressures have no effect on ion pump activity, as it requires several MPa of hydrostatic pressure to inhibit Na^+^-K^+^-ATPase ion pumps via hydrostatic pressure [54, 55]. Given there is adequate blood supply to the eye, and given the operation of ion pumping is independent of physiological achievable pressures within the eye, it is convenient to write Starling force equation for total hydraulic outflow ${\dot{V}}_{i}$ across the *i*^th^ tissue or anatomic structure of the eye (which includes the retinal pigmented epithelium, the non-pigmented epithelium of the ciliary body and the conventional and unconventional outflow pathways) as:

$${\dot{V}}_{i}=\int L_{p}^{}{}_{i}dS_{i}[\Delta p_{i}-\Delta \pi_{i}]=C_{i}^{SL}(p-p_{ref-i})+PF_{i}$$ (4)

where $C_{i}^{SL}=\int L_{p}^{}{}_{i}dS_{i}$ is the hydraulic conductance of the *i*^th^ tissue or anatomical structure through which fluid may exit the eye, *p* is the intraocular pressure, *p*_*ref−i*_ is the ‘membrane reference pressure’ for the *i*^th^ tissue or anatomical structure, *PF*_*i*_ is the outflow at *i*^th^ tissue or anatomical structure due to ion pumping alone. The total pressure dependent outflow (${\dot{V}}_{out}$) for the eye is simply the sum of the outflows across the individual tissues and anatomical structures.

We note it is possible that the effective hydraulic conductance area (*S*_*i*_) and the hydraulic filtration coefficient may change as a function intraocular pressure (e.g. membrane tension may stretch the membrane and increase the effective conductance area or intraocular pressure may alter the size of membrane flow pathways). While we assume here that the membrane area and filtration coefficient are constant, if their variation with pressure is known, this pressure effect can be incorporated in $C_{i}^{SL}$ at this point. Of course the consequences of doing this need to be carefully followed when making model predictions and estimating model parameters.

It is usual to assume that the intraocular pressure is uniform throughout the eye, so we do not need to identify individual intraocular pressures for each tissue or for each anatomical structure. We note that for the case of pressure dependent outflow through the conventional route, we can set pump flow to zero (*PF*_*con*_ = 0), and then rearrangement of Eq (4) recovers the Goldman equation [19, 33, 49].

Here we wish to further develop the theory of pressure dependent outflow facility. This purpose is best served by now defining the local pressure dependent outflow at a specified intraocular pressure using a differential equation. To be specific, we first define the ‘local pressure dependent outflow facility’ of the eye at IOP *p*, denoted here by *C*^*p*^, as the limit as Δ*p*→0 of a ratio of two finite differences, the numerator being the difference in two outflow flow rates at two IOPs, and the denominator being the difference of the two IOPs) [39, 45, 49], which is by definition the derivative of total pressure dependent outflow with respect to IOP. In other words, the local pressure dependent outflow is the tangent to the total outflow curve versus IOP at pressure *p* of interest. Differentiating Eq (4) with respect to *p* and then summing across all *n* tissues or anatomic structures of the eye leads to,

$$C_{}^{p}=\frac{d{\dot{V}}_{out}(p)}{dp}=\sum_{i=1}^{n}\frac{d{\dot{V}}_{out-i}}{dp}=\sum_{i=1}^{n}C_{i}^{SL}\frac{d(p-p_{ref-i})}{dp}=\sum_{i=1}^{n}S_{i}L_{pi}^{}\frac{d(p-p_{ref-i})}{dp}$$ (5)

where *S*_*i*_ is the total surface area of the *i*^th^ membrane normal to the *i*^th^ outflow, and ${\dot{V}}_{out}$ is the total pressure dependent outflow from the whole eye. This means the ‘local pressure dependent outflow facility’ is simply the derivative of the pressure dependent outflow curve with respect to IOP. We immediately see that if all *p*_*ref−i*_ are constants (i.e. the *p*_*ref−i*_ do not change with *p*) this reduces to:

$$C_{}^{p}=C_{T}^{SL}=\sum_{i=1}^{n}C_{i}^{SL}$$ (6)

We could drop the superscript ‘*p*’ in this case and denote this as simply *C* (with units microliters/min/mm Hg), because the local outflow facility is independent of intraocular pressure. More generally though, the various reference pressure *p*_*ref−i*_ are not constant, but are themselves a function of intraocular pressure. We then see that even when $C_{i}^{SL}$ remains constant as intraocular pressure changes, a changing *p*_*ref−i*_ implies that a local of pressure dependent outflow facility at pressure *p* for the *i*th anatomical or tissue structure, $C_{i}^{p}$, may also change, because (*p* − *p*_*ref−i*_) may change with IOP. That is, if we differentiate Eq (4) with respect to intraocular pressure, we find

$$C_{i}^{p}=C_{i}^{SL}\frac{d(p-p_{ref-i})}{dp}$$ (7)

where $C_{i}^{p}$ is the local pressure dependent outflow for the *i*^th^ tissue or anatomic structure. This more complicated expression for $C_{i}^{p}$ occurs because of the way local outflow facility at pressure *p* is defined as the product of $C_{i}^{SL}$ and *d*(*p* − *p*_*ref−i*_)/*dp*, while *d*(*p* − *p*_*ref−i*_)/*dp* is not a constant even when $C_{i}^{SL}$ is constant. The critical point here is the local outflow facility (which is generally not measured) is defined in terms of the change in pressure dependent outflow for a unit change in intraocular pressure, while the biophysics of the outflow actually involves three quantities: the membrane properties (parameter $C_{i}^{SL}$), the intraocular pressure (*p*) *and* the membrane reference pressure (*p*_*ref−i*_). What then might we expect to observe if *p*_*ref−i*_ varies with the intraocular pressure? We now explore the consequences of this, but as described in the Discussion, it is appears likely that multiple tissue processes and their non-linear interactions are responsible for membrane reference pressures increasing with increasing intraocular pressure.

To progress our theory, we now quantitate the difference in the rates of change of intraocular pressure and membrane reference pressure with respect to a change in intraocular pressure (i.e. quantitate *d*(*p* − *p*_*ref*_)/*dp*). This could be done using a variety of approximating functions, some of which may be more or less suitable for particular experimental data, but a reasonable and very mathematically convenient expression involving only one parameter is an exponential decay function that depends on intraocular pressure, viz,

$$\frac{d[(p-p_{ref-i})]}{dp}\approx e^{-\alpha_{i}p}=M_{i}^{p}$$ (8)

where *α*_*i*_ is (normally) a positive coefficient representing the decay rate for the difference in rates of change of intraocular pressure and membrane reference pressure with increasing IOP. We use the approximation symbol (≈) to indicate that the exponential function is an approximation to the actual decay function for an eye tissue or anatomical structure, which may be experimentally estimated within the limits of measurement uncertainty. We mention here that if there is an (additive) constant ‘background fluid pressure’ (say *p*_*B*_) throughout the whole eye system, then this is easily accommodated by introducing another parameter into Eq (8) (viz, $e^{-\alpha_{i}(p-p_{B})}$), but this model is not pursued here.

We now observe from Eq (8) that when *α*_*i*_ is zero or *p*→0, then $M_{i}^{p}$ is one, meaning that at zero IOP there is no change in the membrane reference pressure for a vanishingly small increase in IOP. And if *α*_*i*_ > 0 and *p*→∞ then $M_{i}^{p}\to 0$. $M_{i}^{p}\to 0$ means that the rates of change of IOP and reference pressure with respect to IOP approach equality. Or in other words, $M_{i}^{p}\to 0$ physically means a unit increase in IOP is matched by a unit increase in membrane reference pressure, so at this point the driving pressure difference across the membrane becomes fixed. To say this in another way, when $M_{i}^{p}\to 0$ the ‘pressure dependent outflow’ becomes effectively ‘pressure independent outflow’.

We now define *p*_*Ti*_ to be the ‘intraocular reference pressure’ (not to be confused with *p*_*ref−i*_, which is the ‘membrane reference pressure’) at which there is no pressure dependent flow across the *i*th membrane or structure. If in addition to no flow at *p*_*Ti*_, there is no fluid flow at IOPs less than *p*_*Ti*_, then *p*_*Ti*_ can be viewed as a ‘threshold IOP’ for fluid flow. We can now integrate Eq (8) from *p*_*Ti*_ up to intraocular pressure *p* to find exactly what the driving pressure is at each intraocular pressure for each anatomical structure, viz,

$$\left(p-p_{ref-i})\approx \int_{p_{Ti}}^{p}M_{i}^{p}dp=\int_{p_{Ti}}^{p}e^{-\alpha p}dp=\frac{1}{\alpha_{i}}(e^{-\alpha_{i}p_{Ti}}-e^{-\alpha_{i}p}\right)$$ (9)

We can simply rearrange Eq (9) to calculate the *i*^th^ membrane reference pressure for any intraocular pressure, viz,

$$p_{ref-i}\approx p-\frac{1}{\alpha_{i}}(e^{-\alpha_{i}p_{Ti}}-e^{-\alpha_{i}p})$$ (10)

We also note in passing that differentiating Eq (10) reveals that the rate of change of membrane reference pressure with respect to intraocular pressure is simply $dp_{ref-i}/dp=1-M_{i}^{p}$.

We also mention here that if $C_{i}^{SL}$ is itself behaving as an exponential decay function of pressure in a similar way to $M_{i}^{p}$ for any reason, we can define a second decay coefficient *α*_2*i*_, and it too can be incorporated into $M_{i}^{p}$. This revised $M_{i}^{p}$ could simply involve the sum of two decay coefficients (*α*_*i*_ + *α*_2*i*_), with one decay constant representing the difference in rates of pressure change, while the other decay constant reflects changes in membrane properties with pressure ($C_{i}^{SL}$). While it is most likely that *α*_2*i*_ would have a positive value, it is possible for *α*_2*i*_ to be negative. For example a negative *α*_2*i*_ exponent may occur if over a range of pressures, the effective surface area available for fluid transport increased exponentially with IOP. However we note that if *α*_2*i*_ is defined and used in this theoretical framework, then experimentally measured driving pressures may or may not match theoretically predicted driving pressures. While any discrepancy may provide useful information in and of itself about the eye system behaviour (which may provide one interesting avenue for future research), as mentioned previously, here we develop and apply the theory assuming all $C_{i}^{SL}$ are constants.

We are now in a position to further develop the analysis by substituting Eq (8) in Eq (7) and summing across all *n* tissues and anatomical structures. We define a local pressure dependent outflow facility for the whole eye at pressure *p* to be *C*^*p*^, then

$$C_{}^{p}\approx \sum_{i=1}^{n}C_{i}^{SL}M_{i}^{p}$$ (11)

We now wish to approximate and simplify this Eq (11) as a product of two sums (rather than a sum of products). To do this we use our previously defined hydraulic conductance for the whole eye, $C_{T}^{SL}$ (see Eq (6)) for definition) as one of the two sums, but we also need to introduce a new approximation function $M_{T}^{p}$ such that,

$$C_{}^{p}\approx C_{T}^{SL}M_{T}^{p}$$ (12)

where $M_{T}^{p}$ is given by,

$$M_{T}^{p}=\frac{\sum_{i=1}^{n}C_{i}^{SL}M_{i}^{p}}{\sum_{1=1}^{n}C_{i}^{SL}}$$ (13)

Clearly the function $M_{T}^{p}$ is an average of the individual component exponential decay functions for each tissue or anatomical structure, weighted by the individual hydraulic conductance. To a first approximation $M_{T}^{p}$ may itself be also represented as an exponential decay function, with decay constant for the whole eye defined as *α* (with units (mm Hg)^-1^). We note that as the exponential decay constants for the various tissue or anatomical structures (*α*_*i*_) become more disparate in magnitude, the range of IOPs over which $M_{T}^{p}$ may itself be reasonably represented using a single exponential decay function reduces. Should this problem arise and it be desired to widen this range to make it more practically useful, a different approximating function for $M_{T}^{p}$ may be chosen, or Eq (11) may be employed directly. Indeed, more detailed analyses of whole eye behaviour may well benefit from using Eq (11) directly.

Importantly, having defined the local outflow facility at pressure *p* to be *C*^*p*^, we can now define the average outflow facility over a pressure range *p*_1_ to *p*_2_ as ${\bar{C}}_{p_{1}}^{p_{2}}$ (see Eq (18) for details). We can now evaluate the total pressure dependent outflow for the whole eye (${\dot{V}}_{out}$): (1) which by definition, ${\dot{V}}_{out}$ is equal to the product of the ‘average total pressure dependent outflow’ ${\bar{C}}_{p_{T}}^{p}$ and the apparent intraocular driving pressure (*p* − *p*_*T*_), that is, ${\dot{V}}_{out}={\bar{C}}_{p_{T}}^{p}(p-p_{T})$, and (2) also by definition ${\dot{V}}_{out}$ is equal to the hydraulic outflow conductance for the whole eye, $C_{T}^{SL}$ (microliters/min/mm Hg) and the outflow driving pressure for the whole eye (*p* − *p*_*ref*_), where *p*_*ref*_ is the membrane reference pressure for the whole eye (*p*_*ref*_ is defined quantitatively below) and (3) we also know ${\dot{V}}_{out}$ is equal to the sum of the products of the hydraulic conductance of the *i*^th^ tissue or anatomical structure ($C_{i}^{SL}$) and the individual driving pressure (*p* − *p*_*ref−i*_) and (4) integrating Eq (11) between *p*_*T*_ and *p*, where *p*_*T*_ is the intraocular no-flow pressure. We can now bring together all these four different definitions and levels of approximating the total pressure dependent outflow and the total pressure dependent outflow facility into two equations. For the total pressure dependent outflow equation we have,

$$\begin{array}{l} {\dot{V}}_{out}={\bar{C}}_{p_{T}}^{p}(p-p_{T})=C_{T}^{SL}(p-p_{ref})=\sum_{i=1}^{n}C_{i}^{SL}(p-p_{ref-i})\approx \int_{p_{T}}^{p}C^{p}dp\\ \approx C_{T}^{SL}\int_{p_{T}}^{p}M_{T}^{p}dp\approx \sum_{i=1}^{n}C_{i}^{SL}\int_{p_{Ti}}^{p}M_{i}^{p}dp \end{array}$$ (14)

where to avoid any confusion we state again that *p*_*ref*_ is defined as the membrane reference pressure for the whole eye while *p*_*T*_ is defined as the no-flow intraocular pressure for the whole eye. The equals sign (=) is used for definitions and the approximation sign (≈) for estimates made based on an approximating function (an exponential decay function is used here). Simply rearranging this equation reveals the ‘average total pressure dependent outflow facility’, ${\bar{C}}_{p_{T}}^{p}$ may also be evaluated in a variety of ways, viz,

$${\bar{C}}_{p_{T}}^{p}=C_{T}^{SL}\frac{\left(p-p_{ref}\right)}{\left(p-p_{T}\right)}=\frac{\sum_{i=1}^{n}C_{i}^{SL}(p-p_{ref-i})}{\left(p-p_{T}\right)}\approx C_{T}^{SL}\frac{\int_{p_{T}}^{p}M_{T}^{p}dp}{\left(p-p_{T}\right)}\approx C_{T}^{SL}{\bar{M}}_{p_{T}}^{p}$$ (15)

where

$${\bar{M}}_{p_{T}}^{p}=\frac{\int_{p_{T}}^{p}M_{T}^{p}dp}{\left(p-p_{T}\right)}\approx \frac{\left(p-p_{ref}\right)}{\left(p-p_{T}\right)}$$ (16)

and

$$(p-p_{ref})\approx \int_{p_{T}}^{p}M_{T}^{p}dp=\frac{\sum_{i=1}^{n}C_{i}^{SL}(p-p_{ref-i})}{\sum_{1=1}^{n}C_{i}^{SL}}$$ (17)

We note that *p*_*ref*_ for the whole eye can be calculated from Eq (17). The ratio (*p* − *p*_*ref*_)/(*p* − *p*_*T*_) defines the way the total pressure dependent outflow facility for the whole eye declines from its maximum value at *p*_*T*_. That is, for any *α* > 0, (*p* − *p*_*ref*_)/(*p* − *p*_*T*_) approaches a maximum as *p* → *p*_*T*_ and declines to zero as *p* → ∞. We now apply this theory to the analysis of both physiological and clinical problems involving the estimation of pressure dependent outflow from the eye.

### Theory for the analysis of pressure-volume and pressure-time measurements

An experimental aim may be to measure the average pressure dependent outflow facility over an experimentally convenient intraocular pressure range. The average pressure dependent outflow facility over a pressure range *p*_1_ to *p*_2_, denoted here ${\bar{C}}_{p_{1}}^{p_{2}}$, is the average of the local outflow facilities between pressures *p*_1_ and *p*_2_. This turns out to be a well-known ratio of finite differences [39, 45], viz,

$${\bar{C}}_{p_{1}}^{p_{2}}=\frac{1}{p_{2}-p_{1}}\int_{p_{1}}^{p_{2}}C^{p}dp=\frac{1}{p_{2}-p_{1}}\int_{p_{1}}^{p_{2}}\frac{d{\dot{V}}_{out}}{dp}dp=\frac{{\dot{V}}_{out-2}-{\dot{V}}_{out-1}}{p_{2}-p_{1}}$$ (18)

where ${\dot{V}}_{out-2}$ and *p*_2_ is incremental outflow rate at pressure two, and similarly for the incremental outflow rate at pressure one. If the finite difference is small, Eq (18) can be employed to approximate $C_{}^{p_{1}}$, for as *p*_2_ → *p*_1_ so ${\bar{C}}_{p_{1}}^{p_{2}}\to C_{}^{p_{1}}$. For animal experiments, an incremental volume flow rate into the eye may be held constant until the IOP stabilizes at a new level above the resting IOP, and the average outflow facility over this pressure range is calculated directly using Eq (18). Very occasionally this technique has been employed using *in vivo* human eyes [41].

More often for human eyes, outflow facility is estimated from recordings of a pressure-volume curve and a pressure-time curve for an individual eye. Direct cannulation of the eye is the most accurate method of first volumetrically loading the eye with fluid, and so measuring the pressure-volume curve for the eye (stage one), and then in stage two, cease volumetric eye loading and manometrically measure the pressure-time decay curve [47, 48, 56].

In the clinic, measurements of eye volume changes with change in IOP are much more error prone, with eye volume change usually estimated based on typical corneal shapes and corneal deformations upon application of a standardized loading to the corneal surface. Eye pressure-time curves are generally measured using pneumatonometry [33]. These approaches have the clinical advantage of removing the need to directly cannulate the eye. However a few researchers have directly cannulated the anterior chamber of the human eye immediately prior to cataract surgery, and estimated outflow facility by first volumetrically loading the eye (i.e. stage one, obtaining a subject-specific pressure-volume curve from which the ocular rigidity can be inferred), then in stage two employing manometric measurements to record the pressure-time curve as excess fluid drains from the eye [47, 48, 56].

To estimate outflow facility obtained using intracameral manometric measurements, we need to equate the rate of volume change of the eye as the difference in net rate of pressure independent volume inflow and pressure dependent volume outflow. If we then express the rate of pressure dependent outflow using the average outflow facility between two pressures, we can use this equation to infer ${\bar{C}}_{nt}^{p}$. Let us now do this. We first write the fluid volume balance equation at arbitrary pressure *p* during stage two (i.e. pressure-time recording, when the external volume loading of the eye is zero) as,

$$\frac{d(V_{e}+\Delta V_{e}^{p})}{dt}={\dot{V}}_{pump}-{\bar{C}}_{nt}^{p}(p-p_{nt})=0$$ (19)

where *V*_*e*_ is the reference eye volume at the normotensive intraocular pressure for the resting eye *p*_*nt*_, $\Delta V_{e}^{p}$ is the change in eye volume from this reference state due to some intraocular pressure *p*, and ${\dot{V}}_{pump}$ is the sum of all pressure independent flows across all tissues or anatomical structures in the eye. The net pumped fluid volume is allowed to be a (slowly) varying function of time—but not of pressure.

To recover a suitable equation to analyse the intracameral manometric data, probably the formally correct method would be to take the partial derivative of Eq (19) with respect to *p* (so removing pressure independent terms), and then immediately evaluating a definite integral between the normotensive pressure *p*_*nt*_ and pressure of interest *p* (so including any time dependent terms). Doing this results in Eq (22) directly. However because of the features of the particular function being analysed, it is possible to recover the same equation by taking a limit of a finite difference ratio (as discussed previously). Because this second option is more intuitively appealing and more frequently employed, we describe this approach.

First we write down Eq (19) at two intraocular pressures, *p*_1_ and *p*_2_, viz

$$\begin{array}{l} \frac{d(V_{e}+\Delta V_{e}^{p_{2}})}{dt}={\dot{V}}_{pump}-{\bar{C}}_{p_{nt}}^{p_{2}}(p_{2}-p_{nt})=0\\ \frac{d(V_{e}+\Delta V_{e}^{p_{1}})}{dt}={\dot{V}}_{pump}-{\bar{C}}_{nt}^{p_{1}}(p_{1}-p_{nt})=0 \end{array}$$ (20)

We note that if ${\dot{V}}_{pump}$ is a slowly varying function of time then *p*_*nt*_ also varies slowly in time, but this change is assumed negligible for short duration tests. Subtraction of these two equations leads to,

$${\frac{d\Delta V_{e}}{dt}|}_{p_{2}}-{\frac{d\Delta V_{e}}{dt}|}_{p_{1}}=-{\bar{C}}_{nt}^{p_{2}}(p_{2}-p_{nt})+{\bar{C}}_{nt}^{p_{1}}(p_{1}-p_{nt})$$ (21)

Usually the normotensive state is the dynamic steady-state intraocular reference pressure, and the eye volume at this pressure is then the reference eye volume. Now let *p*_1_ → *p*_*nt*_, then ${\bar{C}}_{nt}^{p_{1}}(p_{1}-p_{nt})\to 0$ and by definition ${\frac{d\Delta V_{e}}{dt}|}_{p_{nt}}\to 0$, which removes these terms. Then changing notation to let *p*_2_ = *p*, and allowing for the possibility of rapid volume injection into the eye (i.e. ${\dot{V}}_{inj}(t)$) perturbing the pressure from the normotensive state (which normally happens in stage one of manometric eye testing i.e. when volumetrically loading the eye with fluid)), we have,

$${\frac{d\Delta V_{e}}{dt}|}_{p}={\dot{V}}_{inj}-{\bar{C}}_{p_{nt}}^{p}(p-p_{nt})$$ (22)

This equation can be used to analyse ‘volume loading’ of the eye (which normally happens in stage one of manometric eye testing). Now setting ${\dot{V}}_{inj}$ equal to zero (which normally happens in stage two of manometric eye testing i.e. when measuring the pressure-time decay curve for the eye), employing the chain rule of differentiation, and using equation (43) (see S1 Supporting Information Ocular Rigidity), we find ${\bar{C}}_{nt}^{p}$ is given by,

$${\bar{C}}_{nt}^{p}=C_{T}^{SL}{\bar{M}}_{nt}^{p}=\frac{{-\frac{d\Delta V_{e}}{dt}|}_{p}}{p-p_{nt}}=\frac{-(\frac{c_{0}}{p}+c_{1}){\frac{dp}{dt}|}_{p}}{p-p_{nt}}$$ (23)

We mention here that an accurate estimation of the ocular rigidity is crucially important for the accurate estimation of average outflow facility between two IOPs from a pressure-time test. Though often not used in clinical practice, employing a pressure dependent ocular rigidity proves particularly important for obtaining accurate estimates of average outflow facility. For this reason we have provided a derivation of pressure dependent ocular rigidity (see S1 Supporting Information Ocular Rigidity), based on the approach developed by Silver and Geyer (2000).

If the average outflow facility and the ocular rigidity are known, then combining Eqs (23), (35) and (38) (see S1 Supporting Information Ocular Rigidity), the rate of change of pressure with respect to time at pressure *p* may be estimated using,

$${\frac{dp}{dt}|}_{p}=-\frac{\hat{K}{\bar{C}}_{nt}^{p}p(p-p_{nt})}{V_{e-nt}}=-C_{c}(p^{2}/p_{nt}-p)$$ (24)

where $C_{C}=\frac{\hat{K}{\bar{C}}_{nt}^{p}p_{nt}}{V_{e-nt}}$ is a parameter (with units min^-1^) governing the pressure-time response behaviour for an individual eye system, and additional variables are defined in S1 Supporting Information Ocular Rigidity. The parameter *C*_*C*_ is analogous to the ‘consolidation coefficient’ governing the consolidation-time response of poroelastic materials, so this parameter may be similarly referred to as a ‘consolidation coefficient’ *C*_*C*_ for the eye. Employing our newly defined *C*_*C*_, a non-dimensional time (*T*) may also be defined as *T* = *C*_*C*_*t*. Then Eq (24) may be written as,

$${\frac{dp}{dT}|}_{p}=(p-p^{2}/p_{nt})$$ (25)

While helpful, because Eq (24) is non-linear, the analytic utility of this approach is probably limited to small changes in pressure. In general, both $\hat{K}$ and ${\bar{C}}_{nt}^{p}$ are functions of pressure, however for small pressure changes, as a first approximation it is common practice to assume they are all independently constants. We note that it is possible for the parameters in *C*_*C*_ to be a function of intraocular pressure independently, but when brought together make *C*_*C*_ more nearly constant. Indeed this happens as cartilage is compressed—the hydraulic conductivity decreases as the stiffness of the cartilage increases with compression—leaving the coefficient of consolidation nearly constant over a significant range of deformation. As ${\bar{C}}_{nt}^{p}$ is a decreasing function with increasing IOP, in the present case, *C*_*C*_ is more nearly constant only when *c*_1_ is negative (see S1 Supporting Information Ocular Rigidity for definition of *c*_1_).

## Results

We begin by first outlining the biophysical meaning of our analysis of pressure dependent outflow for the theory developed above. As a first step we use Eq (14) to plot the total pressure dependent outflow as a function of intraocular pressure, for a range of exponential decay constants. We then plot the average outflow facility versus intraocular pressure (Eq (26)) and the local outflow facility versus intraocular pressure (Eq (27)) for a range of exponential decay constants.

Using published data for the *in vivo* human eye, we then estimate *p*_*T*_ and the range of normal values for the exponential decay constant *α*. We make a ‘first-principles’ estimates of outflow pathway specific parameters (e.g. *p*_*Tch*_ and *p*_*Tcon*_ and *α*_*ch*_ and *α*_*con*_), and then estimate model parameters *p*_*T*_ and *α* for the whole eye. We then estimate *α* and $C_{T}^{SL}$ for both vervet monkey data and for data on enucleated human eyes. Then using published pressure-volume curves, ocular rigidity estimates and pressure-time curves for *in vivo* human eye, we work through Examples 1 to 5, estimating model parameters and outflow facility using Eq (23). Finally, we estimate a range of values for the consolidation coefficient for the *in vivo* human eye, and the total pressure dependent outflows for the *in vivo* eye.

### Total pressure dependent outflow, average and local outflow facilities

Unless otherwise stated, for all the following plots we have assumed the intraocular reference pressure *p*_*T*_ equals 3 mm Hg, while normotensive IOP is assumed to be 15 mm Hg. For all the following plots we arbitrarily assume the hydraulic conductance for the whole eye, $C_{T}^{SL}$ is 1 microlitre/minute/mm Hg (see Eq (6) for definition of $C_{T}^{SL}$).

The driving pressure (*p* − *p*_*ref*_) is calculated using Eq (17) or Eq (26) with *p*_*T*_ equal to 3 mm Hg, or by evaluating the definite integral of the local outflow facility curves from *p*_*T*_ equals 3 mm Hg up to the pressure of interest.

**Fig 1:**
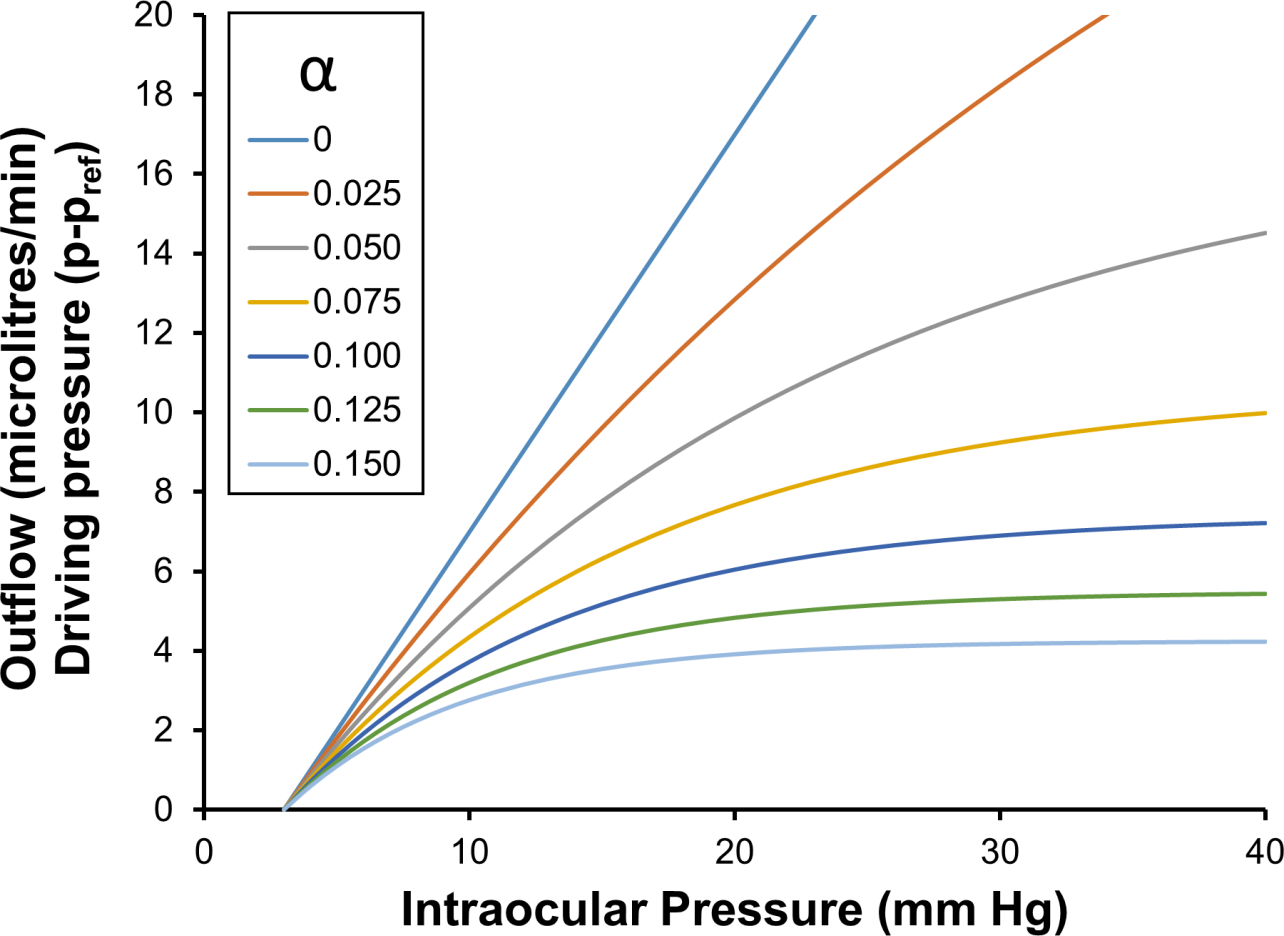
Driving pressure and total pressure dependent outflow as a function of intraocular pressure, for a range of values of *α* (note: $C_{T}^{SL}$ is 1 microlitre/min/mm Hg).

Because the hydraulic conductance for the whole eye ($C_{T}^{SL}$) is chosen to be 1 microlitre/min/mm Hg, the total pressure dependent outflow curves are exactly the same curves as the ‘driving pressure’ (*p* − *p*_*ref*_) curves (see Eq (14))—hence the vertical axis has two separate labels (**Fig 1** and see Eq (17) for definition of (*p* − *p*_*ref*_)). For these chosen values, we can plot the total pressure dependent outflow as a function of intraocular pressure for a range of different decay constants *α* (**Fig 1**). Because $C_{T}^{SL}$ is assumed to be a constant in the theory, we note that total pressure dependent outflows for different values of $C_{T}^{SL}$ can be obtained by simply scaling the outflow curves shown for unitary $C_{T}^{SL}$.

**Fig 1** immediately predicts that if the decay constant is zero, then the total pressure dependent outflow is proportional to the intraocular pressure. The proportionality constant is the average outflow facility, which is the slope of the linear outflow curve for *α* equals zero. We see from **Fig 1** that as the decay constant (*α*) increases the total pressure dependent outflow decreases for any chosen intraocular pressure, and that the rate of increase of total pressure dependent outflow for any constant *α* decreases with increasing intraocular pressure.

If the outflows or the driving pressures are known at two IOPs, **Fig 1** allows *α* be estimated based on the ratio of the outflows or driving pressures. However most importantly, if the driving pressures for the whole eye are known at two IOPs, then it is also numerically possible to estimate both *α* and *p*_*T*_ from the two known driving pressures by simultaneously satisfying the ratio of the driving pressures as well as their magnitudes. If the outflows are also known at the two IOPs and *p*_*T*_ is 3 mm Hg, then it possible to also estimate $C_{T}^{SL}$ by simply scaling the outflows shows in **Fig 1** until it matches the measured outflows (otherwise $C_{T}^{SL}$ can be estimated numerically for any *p*_*T*_).

**Fig 2:**
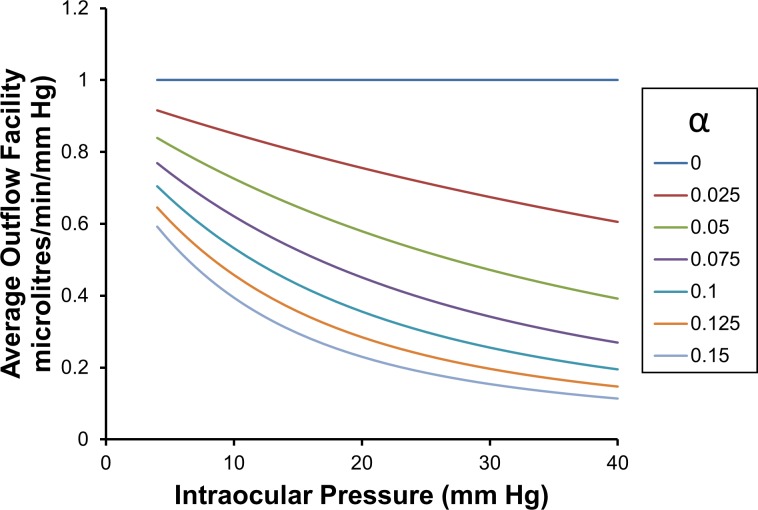
Average outflow facility ${\bar{C}}_{3}^{p}$ as a function of intraocular pressure and for a range of values of *α*.

The average outflow facility is calculated over the intraocular pressure range from *p*_*T*_ to *p* (see Eq (15) or Eq (26)). From these equations the average outflow facility ${\bar{C}}_{p_{T}}^{p}$, can be calculated as shown in **Fig 2**. Examining **Fig 2** it is clear that the curve representing average outflow facilities for curve *α* > 0 approach a maximum as *p*→0. With the exception of *α* = 0, all curves decline towards zero as *p* → ∞.

The average outflow facility shown in **Fig 2** is the average of all the local (or point) estimates of outflow facilities over the intraocular pressure range *p*_*T*_ up to *p*. This average can be calculated by taking the definite integral with respect to intraocular pressure of the local outflow facilities between *p*_*T*_ and *p*, and then dividing by (*p* − *p*_*T*_) (see Eq (18)). Doing this we find for $C_{T}^{SL}=1$,

$${\bar{C}}_{p_{T}}^{p}=\frac{\left(p-p_{ref}\right)}{\left(p-p_{T}\right)}\approx {\bar{M}}_{p_{T}}^{p}=\frac{1}{\left(p-p_{T}\right)}\frac{\left(e^{-\alpha p_{T}}-e^{-\alpha p}\right)}{\alpha }$$ (26)

**Fig 3:**
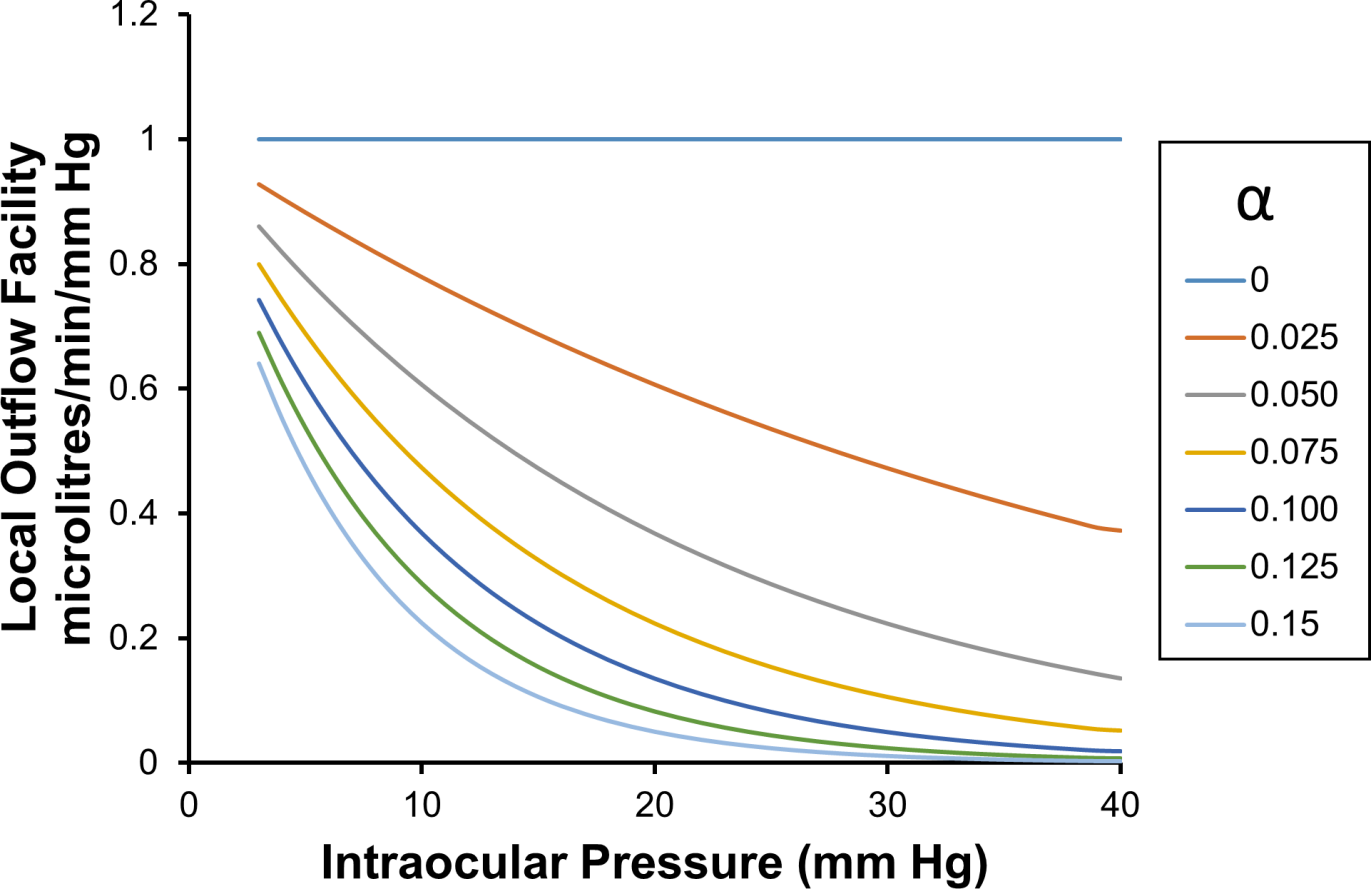
Local (or point) outflow facility *C*^*p*^ as a function of intraocular pressure and for a range of values of *α*. These curves may be calculated either using Eq (27), or by taking the derivative with respect to IOP of the total pressure dependent outflow curves shown in **Fig 1**.

The average outflow facility ${\bar{C}}_{3}^{p}$ is calculated either using Eq (26), or by evaluating the average of the local outflow facility curves shown in **Fig 3** between *p*_*T*_ equals 3 mm Hg and the pressure of interest *p*. The average of the local outflow facility curves can be evaluated by first evaluating the definite integral of the local outflow facility curves shown in **Fig 3**, from 3 mm Hg up to the pressure of interest, then dividing by range of the definite integral i.e. dividing by (*p*−3) mm Hg. We note that the average outflow facility over an ocular pressure range is plotted at the upper limit of the pressure range, that is, at *p*.

Local outflow facilities (see Eq (12)) for $C_{T}^{SL}=1$, are given by

$$C^{p}=M_{T}^{p}=e^{-\alpha p}$$ (27)

and this equation is plotted in **Fig 3**. It is clear from **Fig 3** that when *α* > 0.1, then for all intraocular pressures greater than about 30 mm Hg, local outflow facilities are close to zero. Physically this means a unit increase in IOP is matched by a unit increase in the membrane reference pressure for the whole eye, so at some intraocular pressure there is effectively no further incremental driving pressure across the membrane with increasing IOP (that is Δ(*p* − *p*_*ref*_) → 0), and so there is no further increase in pressure dependent outflow with increasing IOP. In other words, for all practical purposes the total outflow eventually becomes pressure independent above a certain IOP. We notice that the IOP at which this pressure independent state is deemed to occur decreases with increasing *α*.

We may notice that each local outflow facility curve associated with each *α* can be uniquely identified by the ratio of local outflow facilities at two different IOPs (i.e. no two local outflow facility curves have the same local outflow facility ratio for the same two IOPs). When attempting to estimate *α* using local outflow facilities, we see that an estimated *α* is usually more accurate when two local outflow facilities are estimated at widely separated intraocular pressures (i.e. the two IOPs are ‘low’ and ‘high’).

We also note that the average outflow facility when estimated from a normotensive pressure and for $C_{T}^{SL}=1$ is given by,

$${\bar{C}}_{nt}^{p}={\bar{M}}_{nt}^{p}=\frac{1}{\left(p-p_{nt}\right)}\frac{\left(e^{-\alpha_{1}p_{nt}}-e^{-\alpha_{1}p}\right)}{\alpha }$$ (28)

**Fig 4:**
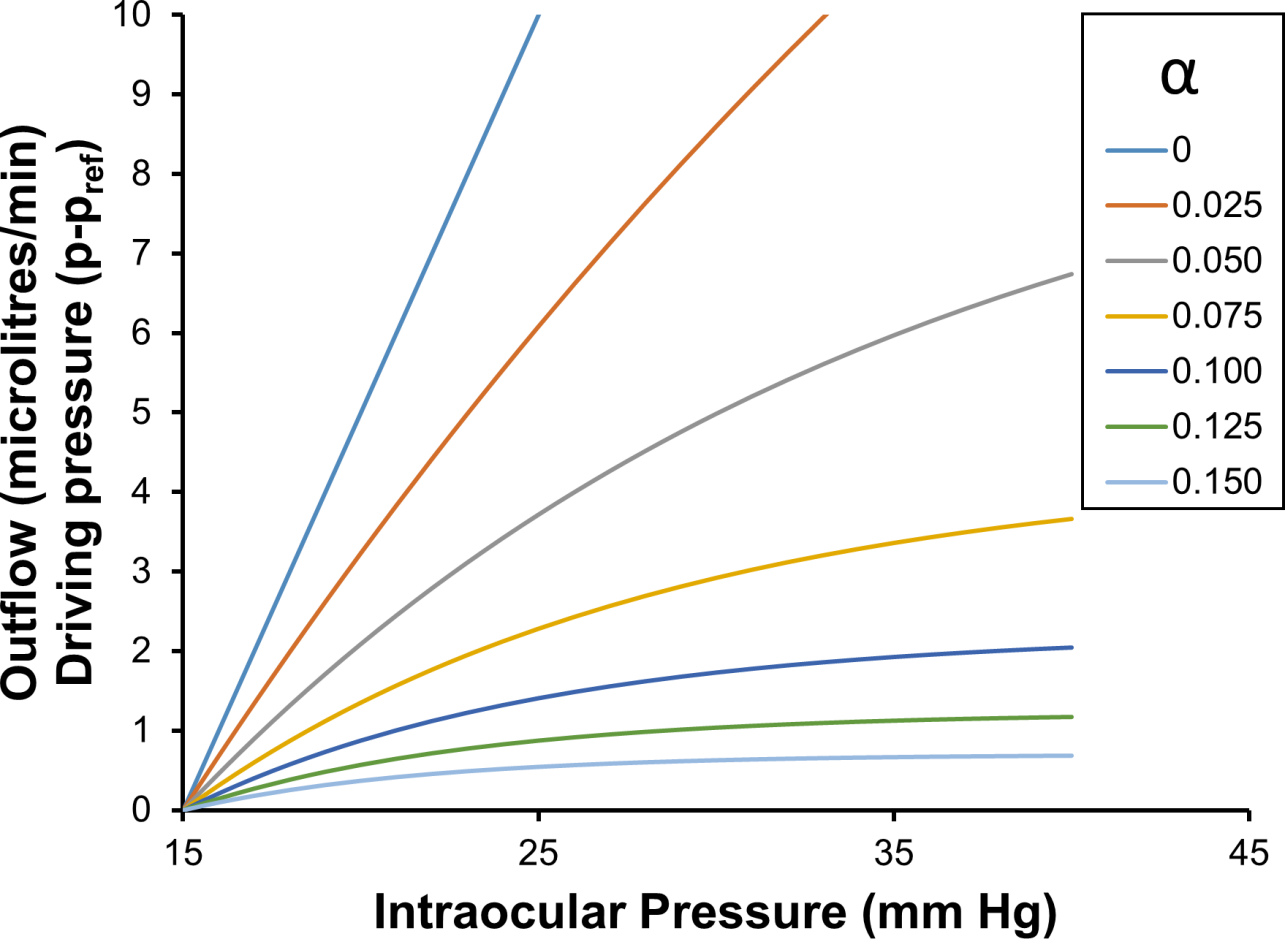
Driving pressure and pressure dependent outflow as a function of intraocular pressure, for a range of values of *α*. Pressure dependent outflows are calculated using Eq (18) with a normotensive pressure of 15 mm Hg as the intraocular reference pressure. The data in this figure can employed to help understand or evaluate Eqs (23) and (24).

**Fig 4** shows the incremental total pressure dependent outflow, found by multiplying Eq (28) by (*p* − *p*_*nt*_). Following ‘volume loading’ of the eye, the average outflow facility **Fig 4** is particularly relevant when estimating the rate of excess fluid leaving the eye during a pressure-time test. Eq (28) is also helpful when attempting to intuitively understand the meaning of the more complicated expressions shown in Eqs (23) and (24).

**Fig 5:**
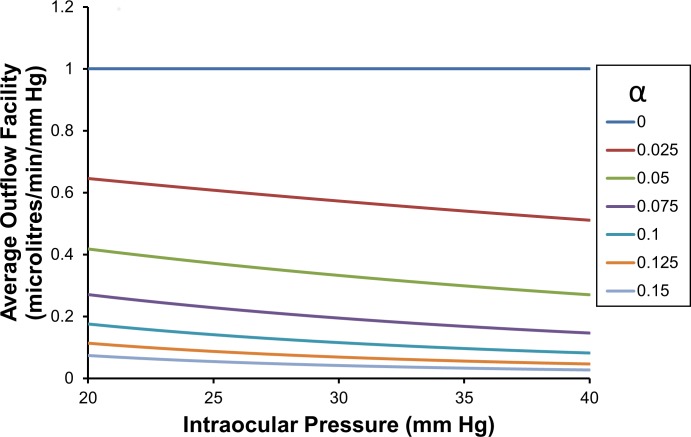
Average outflow facility ${\bar{C}}_{15}^{p}$, as a function of intraocular pressure and for a range of values of *α*. **The average outflow facility** ${\bar{C}}_{15}^{p}$ **is calculated using Eq (28)**. Note: the average outflow facility curves shown in the figure above are not the same as the average outflow facility curves shown in **Fig 2**, as the pressure range over which averaging occurs are different in the two figures. For example for *α* equal to 0.05, at 20 mm Hg the average outflow facility in the above figure is just over 0.4 microlitres/minute/mm Hg, while in **Fig 2** it is about just under 0.6 microlitres/minute/mm Hg. The relative difference in the two average outflow facilities increases with increasing *α*.

**Fig 5** shows the average outflow facility ${\bar{C}}_{15}^{p}$ (Eq (28)), which is what might be expected to be typically estimated in the clinical context (assuming a normotensive eye at 15 mm Hg). It is readily apparent that it appears as though the outflow facility ${\bar{C}}_{15}^{p}$ is reasonably approximated as a linear function of IOP over the pressure range 20 mm Hg to 40 mm Hg, but in fact all the curves are intervals of an (integrated) exponential decay function of IOP divided by (*p* − *p*_*nt*_) (Eq (28)).

The averaging process smears out information, so while it is theoretically and practically possible to estimate the decay constant *α* from at least two average outflow facilities at two different pressure using the ${\bar{C}}_{15}^{p}$ curves (particularly for smaller alphas with ‘steeper slopes’), due to measurement uncertainty, it becomes practically more difficult to do this accurately as *α* becomes larger. When attempting to estimate *α* using average outflow facilities, we note that an estimate is most accurate when two average outflow facilities are estimated with as widely differing averaging pressure ranges as practically possible.

### Effect of assuming an incorrect reference IOP on average outflow facility and outflows for zero threshold pressure

**Fig 6:**
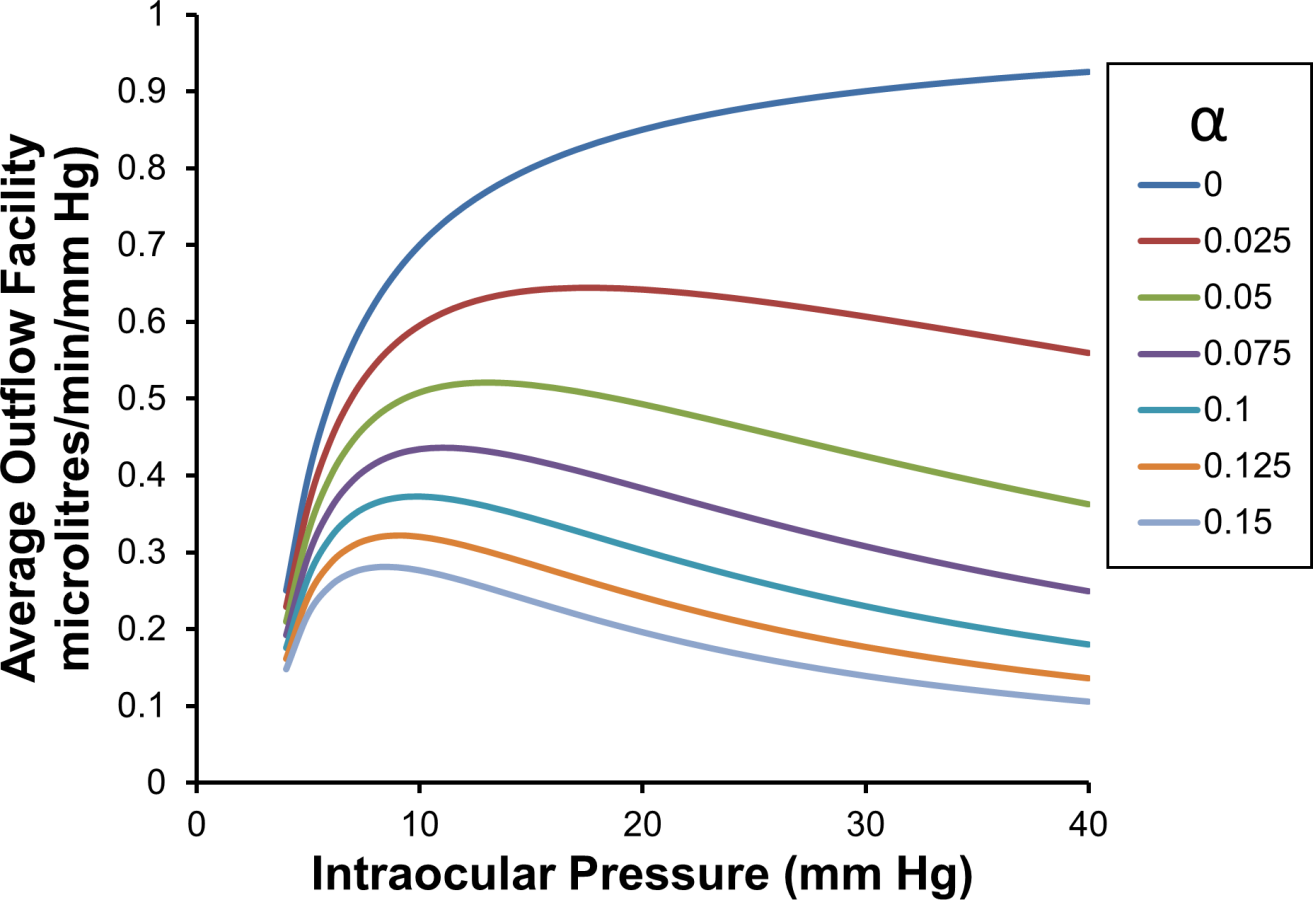
Average outflow facility ${\bar{C}}_{0}^{p}$ as a function of intraocular pressure and for a range of values of *α*. These average facility curves are calculated using Eq (26), but wrongly assumes *p*_*T*_ is equal to zero, when in fact *p*_*T*_ equals 3 mm Hg.

We examine here the situation where we know the actual total pressure dependent outflows, but we assume incorrectly that the reference pressure *p*_*T*_ is zero mm Hg rather than 3 mm Hg (so we then calculate the average outflow facility by dividing by the intraocular pressure *p* rather than (*p* − *p*_*T*_), we obtain quite a different set of curves for average outflow facility to those shown in **Fig 1**. The average outflow facility curves shown in **Fig 6** now appear to have maxima when *α* > 0. We observe that the estimated average outflow facility slowly converges to the correct value as *p* → ∞ and interestingly, at low intraocular pressures the curves converge at the actual no-flow intraocular reference pressure *p*_*T*_. On the other hand the local outflow facilities remain unchanged as they should (see **Fig 3**), which is very important from an experimental viewpoint. The local outflow facility curve is fundamental (there is only one local outflow facility curve, at least for the theory as developed here), while average outflow facility changes with the averaging interval (and any assumptions made about the averaging interval). However local outflow facilities are inherently more difficult to measure, requiring greater measurement accuracy and precision.

**Fig 7:**
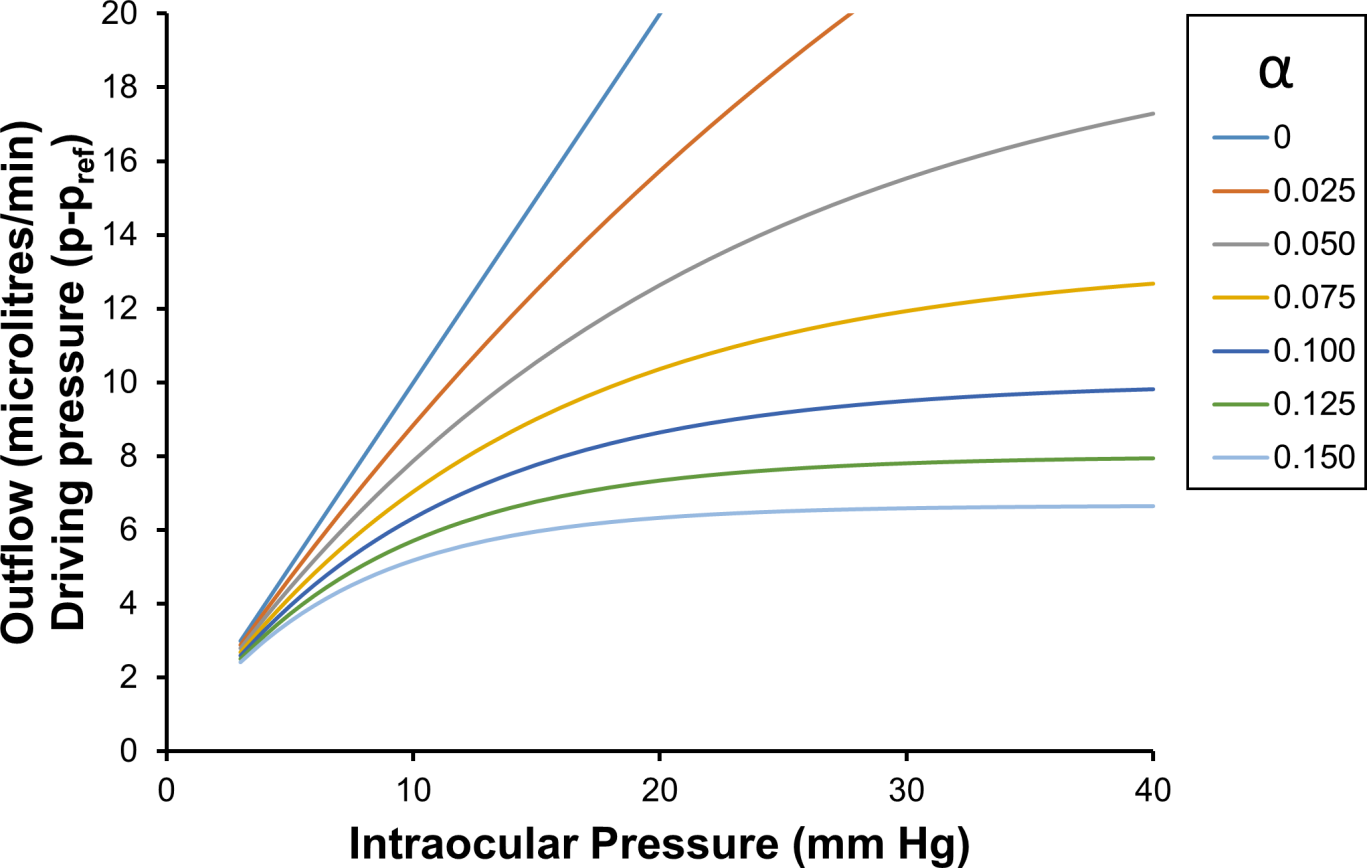
Driving pressure and total pressure dependent outflow as a function of intraocular pressure, and for a range of values of *α*. The driving pressure (*p* − *p*_*ref*_) is calculated using Eq (14) with *p*_*T*_ equal to zero.

**Fig 6** demonstrates clearly that the accurate estimates of the average outflow facility (${\bar{C}}_{p_{T}}^{p}$) and total pressure dependent outflow for the eye depend on knowing the intraocular reference pressure for the eye. In **Fig 7** we show the predicted total pressure dependent outflow when *p*_*T*_ = 0. All predicted total pressure dependent outflows are seen to be translated vertically upwards relative to the outflow curves shown in **Fig 1**. This clearly demonstrates that assuming a no flow reference pressure *p*_*T*_ of zero mm Hg, when in fact it occurs at 3 mm Hg, results in higher estimates of outflow facility than actually occurs (in other words, assuming *p*_*T*_ = 0 do not lead to conservative estimates of total pressure dependent outflow).

### Estimating exponential decay constants for normal *in vivo* eyes from ‘first principles’ based on animal and human data

With this background understanding of pressure dependent outflow predicted by the model, let us now try to estimate plausible values of model parameters for *in vivo* eyes. We initially adopt a ‘first-principles’ modeling approach, meaning we focus on finding experimental values for the fluid driving pressure on both sides of the ‘membrane’ at two different intraocular pressures for two of the major pressure dependent outflow pathways, namely: (i) the retinal pigmented epithelial-choroidal pathway and (ii) the conventional outflow pathway. In the following, we explain in detail the reasoning and all the steps in the calculations leading to parameter estimation, referring to the previous figures where possible to demonstrate various steps. All calculations are done numerically.

We first consider the retinal pigmented epithelial-choroidal pressure dependent pathway, that is, outflow through the retina and across the retinal pigmented epithelium (RPE) (‘RPE cells assembled by tight junctions, forming a continuous epithelium monolayer’), through Bruch’s membrane (‘composed of 5 layers: the basement membrane of the choriocapillaries, an outer collagenous layer, a central elastic layer, an inner collagenous layer, and the basement membrane of the RPE’) to the choroidal interstitial space and finally into the choriocapillaris [57]. As far as the authors are aware, suitable data for the detailing the choroidal interstitial response to changes in intraocular pressure in humans is not available. Similar data for animals is rare. However, fortunately there is one paper reporting quality data obtained from 18 cynomolgus monkeys (see [23]). Fig 5 in Emi et al (1989) shows exactly the driving pressure data required to estimate *α*_*ch*_ (where the subscript *ch* denotes the retinal-epithelial-choroidal flow path).

At very low pressures there is some uncertainty about the accuracy for choroidal interstitial pressure readings obtained via direct cannulation of the choroidal tissue, and so additional pressure readings we made (via cannulation) of a silicone sponge inserted into the choroid. We note that direct cannulation of the choroidal tissue appears to behave more or less as predicted by the present theory at low IOPs (possibly due to a threshold opening pressure for the interstitial space [58, 59] around the choriocapillaris, which would explain the failure of direct cannulation to record very low pressures, rather than experimental artefact). We need to keep in mind too that the sponge does not measure normal choroidal interstitial fluid pressure, rather it measures sponge interstitial pressure in a grossly deformed choroid (see Fig 3 [23]). Surgical trauma associated with sponge insertion may well increase sponge protein concentrations, creating an experimental artefact that significantly influences low fluid pressure readings via osmotic pressure due to the abnormally high protein concentration. Nevertheless the authors provide measurement data over the pressure range from 5 mm Hg to 60 mm Hg by both methods, so appreciating the uncertainty we can assess the data and select a convenient pressure range for analysis. Importantly the authors conclude that: ‘it is clear from the present study that the pressure difference [i.e. pressure between IOP and the pressure in the suprachoroidal space] is significantly correlated to the level of IOP’ [23]. We observe the driving pressure for retinal-choroidal outflow is *not* equal to the IOP minus a constant choroidal interstitial pressure, but instead the driving pressure increases at a decreasing rate with increasing IOP (at least over the pressure range up to 40 mm Hg). Let us try to describe quantitatively how the driving pressure varies by choosing a suitable *α*_*ch*_.

For convenience we choose to calibrate the approximating function to fit the data over the pressure range 3 mm Hg to 40 mm Hg. Over this pressure range we expect that our approximation to plausibly represent the two measured data sets for driving pressure (see Fig 5 [23]). Taking into account the increasing curvature with decreasing IOP, we see that extrapolating the experimental data obtained by direct posterior suprachoroidal cannulation from 5 mm Hg to lower IOPs, that the no flow IOP (*p*_*Tch*_) is plausibly about 3 mm Hg (see Fig 5 [23]). We therefore initially assume that *p*_*Tch*_ equals about 3 mm Hg.

From Fig 5 [23], we see for IOP 10 mm Hg the driving pressure is about 3.2 mm Hg, and at IOP 40 mm Hg the driving pressure is about 7.1 mm Hg. We then take the ratio of the driving pressures at the two IOPs (i.e. 3.2/7.1 = 0.45), and searching for the alpha that has this ratio in **Fig 1** (*α*_*ch*_ is estimated as 0.075). But we see the driving pressures are too high at this alpha, so we would like to adjust *p*_*Tch*_ and find a new estimate of *α*_*ch*_ that has both the right ratio *and* right driving pressures. We do this numerically by trial and error and find the solution satisfying these constraints is *p*_*Tch*_ equals 3.85 mm Hg and *α*_*ch*_ equals 0.095. These parameters provide a reasonable fit to the driving pressure data over the range 5 to 50 mm Hg. We now turn our attention to pressure dependent outflow through the anterior chamber of the eye.

There are two anterior pressure dependent flow paths reported in the literature: the so-called conventional (trabecular meshwork) pathway that is pressure dependent, and the so-called unconventional (uveoscleral) pathway, which is believed to be pressure independent [7]. We therefore focus on the conventional pressure dependent pathway (denoted by subscript *con* in the following), and we focus on the driving pressure between the IOP and the episcleral venous pressure, which ‘can be described as a back-pressure against which aqueous humor must flow’ [60].

The episcleral venous pressure in humans (denoted EVP in the text, and by subscript *evp* in mathematical symbols) is reported to be normally in the range 7 to 10 mm Hg when the intraocular pressure is 15 mm Hg in the upright position [51, 60–62]. The intraocular no-flow pressure could be interpreted as the membrane reference (back) pressure in the no flow state, but this pressure is uncertain for the conventional outflow pathway. One initial approach to guide estimation of *p*_*Tcon*_ is to look at the ‘orbital venous pressure’, which is remote from the episcleral venous pressure that is normally measured close to the limbus. The ‘orbital venous pressure’ is reported to be about 2 to 3 mm Hg in rabbits at normal mean blood pressure [63]). Another indicator of a suitable reference back-pressure pressure indicative of *p*_*Tcon*_ for the rhesus monkey, is the episcleral venous pressure becoming independent of IOP at a distance of ‘about 4 to 5 mm from the limbus, with the tip of the cannula directed away from the eye’ [25]. Brubaker (1970) reported a minimum such episcleral venous pressure of 3.1 mm Hg for rhesus monkeys fitted with neck bands (see animal 7 listed in Table I [25]). On the basis of this animal data, we again adopt *p*_*Tcon*_ equal to 3 mm Hg as being a reasonable first guesstimate.

We therefore assume that the membrane pressure reference increases from 3 mm Hg to 8 mm Hg EVP as the intraocular pressure changes from 3 to 15 mm Hg (so (*p* − *p*_*evp*_) = 7 mm Hg). We can also estimate a lower bound pressure reference change to be from 3 mm Hg to 6 mm Hg as intraocular pressure changes from 3 mm Hg to 15 mm Hg (so (*p* − *p*_*evp*_) = 9 mm Hg), and we can estimate the upper bound reference pressure change to be from 3 mm Hg to 10 mm Hg as intraocular pressure changes from 3 mm Hg to 15 mm Hg (so (*p* − *p*_*evp*_) = 5 mm Hg). We note Acott et al (2014) reports that the driving force between the IOP and the episcleral veins is around 6 mm Hg to 9 mm Hg for the normal eye [51], which is close to the range estimated above (i.e. 5 mm Hg to 9 mm Hg).

We now have initial information about *p*_*Tcon*_ and the outflow driving pressure at one IOP, but to unequivocally estimate *p*_*Tcon*_ and *α*_*con*_, we actually need additional information. For example when estimating *α*_*ch*_, we took the ratio of two driving forces at two known IOPs (eliminating the hydraulic conductivity) and sought to optimize the driving pressures and their ratio by adjusting *p*_*Tcon*_ and *α*_*con*_. But in the present case, we do not have the driving forces at two IOPs, rather we have the driving force at only one IOP. Hence we propose to proceed without sufficient information, but then check our estimate with independent information. That is we use independent experimentally measured $C_{con}^{SL}$ and outflow facility or known total outflow to confirm the accuracy of a proposed estimate. If it is not accurate, we will iterate to find a better solution. Adopting this strategy, we assume *p*_*T*_ equals 3 mm and as an initial guess we read *α*_*con*_ directly from **Fig 1** at 15 mm Hg. We find for the known driving pressure of 7 mm Hg at an IOP of 15 mm Hg, our initial guess for *α*_*con*_ is about 0.0625.

We now independently check the accuracy of our estimate. We can proceed in two ways: (i) check the total outflow, and (ii) check the outflow facility. We check both ways using two independent sets of data. But because it is useful to us in the following, we first calculate the hydraulic conductance of the conventional route by dividing the ‘typical [measured pressure dependent] human outflow rates’, which is equal to 2.5 to 3.0 microlitres/min [51] (and we mention in passing that the lower end of the range of outflow rates are more likely in people > 65 years [64]) by the median estimate for the driving pressure (i.e. 7 mm Hg) to estimate the hydraulic conductivity for the conventional pathway as $C_{con}^{SL}=0.36-0.43$ microlitres/min/mm Hg. We note that for 23 human anterior segment organ culture experiments, [51] reports $C_{con}^{SL}=0.34$ microlitres/min/mm Hg, just outside the lower end of our estimated range.

Now our first and simplest method is to check the predicted total outflow with measured total outflow *in vivo*. We multiply the outflow at 15 mm Hg, unit hydraulic conductance and *p*_*Tcon*_ equals 3 mm (i.e. 7.0 microlitres/min for alpha equals 0.0625) by $C_{con}^{SL}$ equals 0.36, to find a predicted outflow of 2.5 microlitres/min. This agrees closely with the independently measured flow rate of 2.4 ± 0.6 microlitres/min reported in [64] for eyes greater than 60 years of age. Based on the agreement between the measured outflow and driving pressure at 15 mm Hg and predicted outflow and single driving pressure 15 mm Hg, we accept *α*_*con*_ is about 0.0625, $C_{con}^{SL}$ equals 0.36 and *p*_*Tcon*_ is assumed to be 3 mm (summarized as Entry 1 in Table 1).

**Table 1:** Data employed for ‘first-principles’ estimation of *p*_*T*_ and *α*_*con*_, which describe the no-flow IOP and the rate of decrease in local pressure dependent outflow via the conventional route.

	Expected (mm Hg)	Lower Bound (mm Hg)	Upper Bound (mm Hg)
*p*_*Tcon*_	3.0/2.5(2.5,7.5)/0.0/0.0	3/3/0/0	3/3/0/0
*p*_*epv*_	8.0/8.0(22.5,26)/6.3/8.0	6/6.7/3.75/6	11/11.7/9.3/11
*p*	15/15 (33,33)/15/15	15/15/15/15	15/15/15/15
(*p* − *p*_*epv*_)	7.0/7.0(10.5,7.0)/8.7/7	9/8.3/11.25/9	4/3.3/6.3/4
(*p* − *p*_*epv*_)/(*p* – *p*_*Tcon*_)	0.58/0.55/0.58/0.47	0.75/0.69/0.75/0.6	0.42/0.28/0.42/0.33
$C_{con}^{SL}$	0.36/na/0.38/0.36	0.36/na/0.38/0.36	0.36/na/0.38/0.36
Flow rate (μl/min)	2.5/2.5(3.5,2.5)/3.3/2.5	3.2/3.3/4.5/3.2	1.4/1.3/2.4/1.4
*α*_*con*_	0.0625/0.07/0.08/0.12	~0.03/0.04/0.04/0.075	~0.13/0.16/0.13/0.25

The second checking method looks at outflow facilities in enucleated eyes. In other words we can multiply the average outflow facility 0.65 microlitres/min/ mm Hg (obtained numerically at IOP equals 15 mm Hg, with *p*_*T*_ = 0 and *α*_*evp*_ = 0.0625) by 0.36 to 0.43 (see above), to obtain a model estimated conventional outflow facility for the *in vivo* normal eye to be in the range 0.23 to 0.28 microlitres/min/ mm Hg. We now compare this predicted outflow facility with measured outflow facilities for *enucleated* human eyes at 15 mm Hg. For 5 donor eyes (mean age 62 years, range 32 to 74 years) the mean outflow facility was found to be 0.21 ± 0.03 microlitres/min/mm Hg [65] (note: implied flow rate 3.2 microlitres/min). In another study, the mean outflow facility for six enucleated human eyes at 15 mm Hg was found to be 0.24 microlitres/min/ mm Hg [44] (note: implied flow rate 3.6 microlitres/min).

It appears that the predicted outflow facilities (range 0.23 to 0.28 microlitres/min/mm Hg when *α*_*con*_ = 0.0625 and *p*_*T*_ = 0) is above the measured range for outflow facilities. So we need to iterate i.e. guess another *α*_*con*_, and follow the same procedure in an attempt to find a better solution. Iterating we find that when *α*_*con*_ = 0.08 the predicted outflow facilities range from 0.21 to 0.25 microlitres/min/ mm Hg, which is in close agreement between the measured range of outflow facilities (range 0.21 to 0.24 microlitres/min/mm Hg). This provides us with a second estimate for *α*_*con*_ based on data obtained from enucleated eyes (summarized as Entry 3 in Table 1).

As explained above, to unequivocally estimate *α*_*con*_ and *p*_*Tcon*_ when both are unknown, parameter estimation is most straight-forward when two driving pressures at two IOPs are known. We have one known driving pressure at 15 mm Hg, but it would be most convenient if there was data for the driving pressure at a higher IOP. Fortunately altering the angle of body tilt and measuring EVP and IOP is one way to obtain such data for the *in vivo* human eye. One experiment measured episcleral venous pressure and intraocular pressure upon inversion (i.e. in the head-down vertical position). Such an experiment was performed on 11 volunteer subjects aged 24 to 38 years of age, with IOP and EPV measured in both the supine and inverted positions [66]. Friberg et al (1987) reports that commencing measurements 1 minute after this postural change, the Pearson correlation coefficient (i.e. slope of regression line) between the measured change in EVP and the measured change in IOP is 0.83 ± 0.21, and that IOP approximately doubles in the inverted position relative to normal IOP. We now analyse this data to estimate the driving pressure at the steady-state IOP in the inverted position.

Employing the reported initial mean IOP of 16.8 mm Hg and a final mean IOP of 32.9 mm Hg [66], and using the abovementioned line slope between change in EVP and change in IOP, EVP rises by (32.9–16.8) × 0.83 = 13.4 ± 3.4 mm Hg. Adding this change in EVP to the initial EVP (assumed to be 9.0 mm Hg at an IOP of 16.8 in the supine position) gives an EVP of 22.4 mm Hg when inverted. The driving pressure (*p* − *p*_*evp*_) at 32.9 mm Hg is then (32.9–22.4) = 10.5 mm Hg. At 15 mm Hg the driving pressure has been estimated above at 7 mm Hg. So the ratio of the two driving pressures at 15 mm Hg and 32.9 mm Hg is about 7/10.5, which equals 0.67. Then searching for the combination of variables that gives the right ratio and magnitude of driving pressures, we find this occurs when *p*_*Tcon*_ = 2.5 mm Hg and *α*_*con*_ is 0.07 (range 0.055 to 0.10) (summarized as Entry 2 in Table 1)

Can the theoretical model tell anything more about the inversion experiment? Given *p*_*Tcon*_ = 2.5 mm Hg and using other model parameters appropriate for the inversion experiment, the model leads to a predicted pressure dependent outflow at 33 mm Hg about (10.5/7) 50% larger than at 15 mm Hg. Given that changing neurohormonal activity can change aqueous production, this seems plausible upon immediate head inversion under normal conditions. On the other hand aqueous production is reportedly unchanged with changes up to ± 50 degrees head position [67], but these outflow measurements were made 30 minutes after the posture change, which presumably gives sufficient time for adaption processes at various levels to occur. Assuming sufficient time has elapsed during the inversion experiment for aqueous production to return to its former rate, in terms of the model developed here, it seems likely the intraocular no-flow reference pressure *p*_*Tcon*_ has changed, possibly driven by changing neural activity in response to the now substantial constant theoretical hydrostatic head in the inverted position. A 30 cm of head of water in an upright tube generates about 22.5 mm Hg of pressure, which we note to be almost identical to our estimated EVP above. Indeed it is presumably this EPV pressure that drives aqueous and blood from the eye back to the level of the heart. Searching for a new *p*_*Tcon*_ that results in the same pressure dependent outflow at 33 mm Hg, it is found by trial and error that if *p*_*Tcon*_ = 7.5 mm Hg in the theoretical model, then the pressure dependent outflow at 33 mm Hg in the inverted position is very nearly unchanged from the pressure dependent outflow at 15 mm Hg in the sitting position (and the driving pressure returns to 7.0 mm Hg). In other words, the model predicts that if the pressure dependent outflow is unchanged, head inversion increases *p*_*Tcon*_ by about 5 mm Hg relative to the sitting position. This change in *p*_*Tcon*_ is much less than the nominal hydrostatic pressure change associated with inversion (about 22.5 mm Hg), but this does suggest that *p*_*Tcon*_ is variable (possibly under neural/hormonal/paracrine controls, which possibly influence cell contraction).

We also mention that if *α*_*con*_ for the eye is known, then the slope of the predicted correlation relating the change in EVP to a change in intraocular pressure between any two IOPs of interest can be evaluated directly by taking the average of $dp_{epv}/dp=1-M_{p_{1}}^{p_{2}}$ over the pressure range of interest. Using the data for the inversion experiment as an example, if it is first known that *α*_*con*_ is 0.07, then we predict the slope of the line relating the change in EVP to change in intraocular pressure between initial mean IOP of 16.8 (approximated as 17) mm Hg and the final mean IOP of 32.9 (approximated as 33) mm Hg. Performing this averaging of $dp_{epv}/dp=1-M_{epv}^{p}$, we find predicted mean line slope is (1 –(0.304–0.99)/(0.07*(33–17))) = 0.82, while the mean slope of the regression line and SEM reported by [66] is 0.83 ± 0.21. The predicted and measured slopes agree closely, again suggesting 0.07 is a good estimate for *α*_*con*_.

We also note that if the change in IOP with body tilt is small (i.e. IOP changes a few mm Hg), then for known *α*_*con*_ the predicted regression slope relating the change in EVP to a change in intraocular pressure can be estimated using **Fig 3**, as 1 minus the local outflow facility (see **Fig 3**) at the intraocular pressure of interest. So for example, if *α*_*evp*_ is 0.075, then at 10 mm Hg IOP the predicted local regression slope relating the change in EVP to a change in intraocular pressure is (1–0.62) = 0.38, at 15 mm Hg IOP the predicted local regression slope is (1–0.53) = 0.47, and at 20 mm Hg IOP the predicted local regression slope is (1–0.45) = 0.55. As expected, all these local regression slopes are much less than the experimentally measured group average regression slope found by [66], because Friberg et al (1987) found an average over a much higher pressure range (initial mean IOP of 16.8 mm Hg and final mean IOP of 32.9 mm Hg, correlation slope 0.83 ± 0.21). Such model predictions point to the potential utility of the model’s predictions for experimental design [68].

We summarise the data and assumptions employed above in estimation of *α*_*con*_ in Table 1. Each cell in Table 1 has four entries (1/2/3/4), identifying four different sets of experimental circumstances and so modelling assumptions to estimate conventional outflow parameters. For example, Entries 1 in the second column assumes *p*_*T*_ is 3 mm Hg, the driving pressure for outflow is 7 mm Hg at an IOP of 15 mm Hg and $C_{con}^{SL}$ = 0.36 microlitres/min/mm Hg). Then to match an experimentally measured outflow of 2.5 microlitres/min we estimate *α*_*con*_ to be 0.0625.

Entries 2 in the second column estimates both *p*_*T*_ and *α*_*con*_ from known driving pressures at two IOPs. The driving pressure is 7 mm Hg at 15 mm Hg IOP and shown in brackets, the driving pressure is 10.5 mm Hg at 33 mm Hg IOP. We estimated *α*_*con*_ to be 0.075 and *p*_*Tcon*_ = 2.5 mm Hg (see details above). Entries 3 in the second column assumes *p*_*T*_ is 0 mm Hg and the outflow facility (*p* − *p*_*epv*_)/(*p* − *p*_*T*_) remains constant (i.e. the same as Entry 1) (see analysis above of enucleated eyes). In other words the third model assumes ${\bar{C}}_{p_{T}}^{p}$ is held constant and *p*_*T*_ is 0 mm Hg—then the flow rate then increases about 38%. Entries 4 in the second column assumes *p*_*T*_ is 0 mm Hg and the driving pressure (*p* − *p*_*epv*_) remains constant (i.e. the same as Entry 1). In other words, the fourth model assumes the flow rate is held constant when *p*_*T*_ is 0 mm Hg, but we notice the estimates for *α*_*con*_ are then much higher than for Entries 3 (the fourth model is not described in more detail here).

We now assess the validity of these four conventional pathway outflow models. The first model estimates *α*_*con*_ knowing one driving pressure at one IOP, and matching the experimentally measured outflow. Because all data is for the human eye, with driving pressure and outflow data for the *in vivo* human eye, this estimate should be reliable. The second model is based on ratio of two driving pressures, so this estimate is theoretically rigorous ($C_{con}^{SL}$ do not influence the estimation of *α*_*con*_ and *p*_*T*_ using this method), so this estimate should also be reliable. Assuming constant outflow facility, the third model predicts a 38% increase in flow rates at 15 mm Hg (range 3.4 to 4.2 microlitres/min) for an enucleated eye compared to the *in vivo* eye. We note that the implied flow rate for Ren et al (2013) is 3.2 microlitres/min, and for Hashimoto and Epstein (1980) is 3.6 microlitres/min. This range (3.2 to 3.6 microlitres/min) is in reasonable agreement with the predicted flow rate range. Indeed, the measured flow rates are nearly equal to the predicted flow rate if the *in vivo* flow is 2.5 microlitres/min and *p*_*T*_ equals 3 mm Hg, so this also appears to be reasonably reliable. Finally the fourth model assumes outflow rate is constant, and this results in a very large predicted *α*_*con*_. Because the flow rates are in fact significantly increased for the enucleated eye, this fourth model is rejected. Indeed, the predictions for *α*_*con*_ in the third model also turn out to be much larger than all later predictions for *α*, which provides confirmatory evidence to reject the fourth model.

With this wealth of experimental data and model estimates, we are now in a strong position to again examine the validity of choosing *p*_*Tcon*_ is 3 mm for the *in vivo* model. Based on Entries 1 and 3 in Table 1, we now have estimated *α*_*con*_ as being about 0.0725 and $C_{con}^{SL}$ (around 0.36 to 0.43). So we can now adjust *p*_*Tcon*_ and compare the prediction pressure dependent outflow at 15 mm Hg with an independently experimentally determined *in vivo* aqueous known flow rate, such as that reported by (e.g. [21], who reports an aqueous outflow of 2.4 ± 0.6 microlitres/min). By this method, we can fix *p*_*Tcon*_.

When we do this, we find for *p*_*Tcon*_ equal to 1 mm Hg, the pressure dependent outflow through the conventional route is 3.3 to 3.9 microlitres/min (i.e. mean 3.6 ± 0.3 microlitres/min), for *p*_*Tcon*_ equal to 3 mm the pressure dependent outflow through the conventional route is 2.3 to 2.8 microlitres/min (i.e. mean 2.55 ± 0.25 microlitres/min), and for *p*_*Tcon*_ equal to 5 mm the pressure dependent outflow through the conventional route is 1.8 to 2.1 microlitres/min (i.e. mean 1.95 ± 0.15 microlitres/min). Comparing these ranges with measured flow rate of 2.4 ± 0.6 microlitres/min [21], *p*_*Tcon*_ equals 3 mm is a clearly a good estimate (the exact value to give 2.4 microlitres/min when $C_{con}^{SL}$ equals 0.36 is *p*_*Tcon*_ equals 2.7 mm Hg).

Finally, to predict the behaviour of the whole eye requires us to find the weighted average of the two estimates for *α*_*i*_ (i.e. *α*_*ch*_ = 0.095 based on cynomolgus monkey data and *α*_*con*_ = 0.063,0.07,0.08 based on reliable human data), and the weighted of average of two estimates of *p*_*Ti*_ (i.e. *p*_*Tch*_ = 3.85 based on cynomolgus monkey data and *p*_*Tcon*_ = 2.5,2.7 mm Hg based on reliable human data). Theoretically one should now use this data to estimate a weighted average (using hydraulic conductivities as the weights) to obtain a suitable *α* and *p*_*T*_ for the whole eye (see Eq (12)), but in this case the reliability of the estimates is probably the dominant issue. Given the human data for the conventional outflow pathway is much more reliable than estimates based on cynomolgus monkey data, we therefore assume that *α* for the whole eye equals about 0.07 (our estimated range is from 0.04 to 0.13), and *p*_*T*_ is taken to be equal to 3 mm Hg. Assuming the above estimates were of the equal reliability these estimates would vary as the relative hydraulic conductivities (or approximately, as fractions of net fluid production exit via the anterior, posterior and vitreous chambers), and then based on the above data *α* and *p*_*T*_ would be somewhat larger than the values chosen above. We note that any initially assumed fractional outflow can be checked once estimates of total pressure dependent outflows have been made, and refinements to the estimates can be made by iteration.

### Estimating pressure dependent outflow parameters for *in vivo* Vervet monkey eyes

The outflow facility (${\bar{C}}_{p_{0}+2.5}^{p_{0}+11.9}$) for Vervet monkeys (n = 45) is reported by [39] to be 0.55 microlitres/minute/mm Hg. ‘Resting intraocular pressure’ (denoted *p*_0_ in Barany (1964)) for Vervet monkeys is approximately 9.0 mm Hg. Barany (1964) considers the effect of an exponentially varying *time* dependent change on outflow facility while testing an animal, but he does not consider an exponentially dependent variation with intraocular pressure on outflow facility. In fact, Barany suggests that there is no pressure dependent change in outflow facility, though he does acknowledge and presciently cautions that data shown in Fig 7 of Barany (1964) may in fact suggest that the outflow facility probably is not constant as the intraocular pressure is lowered (‘Perhaps at these low pressures the outflow channels are not always filled with aqueous in a normal manner and C therefore not constant under changing pressure’ [39]). Careful consideration of the data shown in Fig 7 of Barany (1964) is indeed consistent with *α* > 0.

This can be seen by calculating the actual ${\bar{C}}_{p_{0}}^{p_{0}+11.9}$ for individual animals based on removing differences in measured and estimated *p*_0_ (i.e. using Eq 2 in Barany (1964) to analyse the data shown in Fig 7), and then plotting measured *p*_0_ against ${\bar{C}}_{p_{0}}^{p_{0}+11.9}$. It is found that ${\bar{C}}_{p_{0}}^{p_{0}+11.9}$ is pressure dependent across the group of animals. An analysis of this plot based on the data shown in Fig 7 of Barany (1964) indicates to a reasonable approximation that *α* is actually around 0.05, rather than zero, and that $C_{T}^{SL}=1.0$. We find when *p*_0_ = *p*_*T*_ is 9.1 mm Hg and $C_{T}^{SL}=1.0$, then ${\bar{C}}_{p_{0}}^{p_{0}+11.9/2}$ is 0.55 microlitres/min as reported by Barany (1964), and the mean outflow (estimated at *p*_0_ + 2.5 mm Hg and *p*_0_ + 11.9 mm Hg) is 3.9 microlitres/min, while Barany (1964) reports 4.0 microlitres/min. This new estimate of *α* has the effect of raising the low estimates of *p*_0_ relative to the measured values reported at lower intraocular pressures, as shown in Fig 7 of Barany (1964). This estimated *α* also explains the somewhat higher outflow facility estimates relative to the measured values at higher intraocular pressures. Taken together this data leads us to estimate ${\bar{C}}_{3}^{p}=C_{T}^{SL}{\bar{M}}_{3}^{p}=1.0{\bar{M}}_{3}^{p}$ microliters/min/mm Hg. At a normal intraocular pressure of 9.1 mm Hg, we can then estimate the average total pressure dependent outflow as ${\bar{C}}_{3}^{9.5}(p_{9.5}-p_{3})=0.73(9.1-3)=4.45$ microlitres/min, and at 15 mm Hg we estimate the total pressure dependent outflow as ${\bar{C}}_{3}^{15}(p_{15}-p_{3})=0.64(15-3)$ equals 7.7 microlitres/min.

### Estimating pressure dependent outflow parameters for enucleated human eyes

Brubaker (1975) reports testing ten, enucleated human eyes (no age details provided). All eyes were studied within 24 hours of enucleation. At 15 mm Hg, testing revealed a mean total pressure dependent outflow to be about 5.9 microlitres/min (see Fig 2 of [43]). However tests on enucleated eyes are less likely to be representative of *in vivo* behaviour (e.g. back-pressure is only generated by distortion of aqueous and vascular networks in the absence of normal blood flow through normal aqueous and vascular networks that have normal autonomic and vascular autoregulation). But exactly how much less representative is uncertain. It is interesting though, to note that total pressure dependent outflow shown in Fig 2 of Brubaker (1975) clearly indicates that outflow facility decreases with increasing intraocular pressure. Indeed if Fig 2 of Brubaker (1975) (exact values shown in Fig 2 of Brubaker (1975) can be computed using the data shown in Table 1 of Brubaker (1975)) is fitted to an exponential decay curve using our model assuming *p*_*T*_ is 2 mm Hg, the mean data for ten eyes indicates *α* ≈ 0.029 (range 0.023 to 0.036) and $C_{T}^{SL}\approx 0.57$ microliters/min/mm Hg. Again *p*_*T*_ equal to 2 mm Hg is in reasonable agreement with an important assumption underlying our later *in vivo* analysis. We also mention that another model, with *p*_*T*_ equal to zero, estimates *α* ≈ 0.02 and $C_{T}^{SL}\approx 0.45$ microliters/min/mm Hg. This second model fits the Brubaker (1975) data with comparable accuracy to the first model. This second model almost exactly approximates Brubaker’s reported slope for the ‘fractional increase in outflow resistance *Q*’ (where *Q* = *d*(*R*/*R*_0_)/*dp* = 0.012) for his linear regression analysis of ‘outflow resistance’ versus IOP (see Fig 3 of Brubaker (1975)). We also observe that assuming *p*_*T*_ is 3 mm, leads to model estimates *α* ≈ 0.038 (range 0.030 to 0.045) and $C_{T}^{SL}\approx 0.70$. This third model is less accurate than the models one and two, but remains well within ± 1 SEM, and serve to illustrate parameter estimates when *p*_*T*_ is 3 mm Hg.

### Estimating pressure dependent outflow parameters for *in vivo* human eyes

Langham and Eisenlohr (1963) measure *in vivo* outflow facility for normal eyes in two subjects (age not specified), by simultaneously measuring the steady-state incremental pressure dependent outflow rate and IOP. Data on incremental outflow rate and incremental IOP is provided at two pressures for one of these two subjects (as shown in Fig 7 of their paper [41]). Assuming the normotensive pressure is 15 mm Hg and *p*_*T*_ equals 3 mm Hg, we can interpret their data using **Fig 5** above. We first estimate *α* from the ratio of the measured average outflow facilities. The average outflow facility between 15 mm Hg and 22 mm Hg is 2.1/7 = 0.30, and between 15 mm Hg and 35 mm Hg is 4.4/20 = 0.22. The *α* in **Fig 5** associated with the ratio 0.22/0.3 at the two specified IOPs is 0.05. Knowing **Fig 5** is constructed for $C_{T}^{SL}=1.0$ we can scale the outflow shown in **Fig 5** to agree with the experimentally measured outflows. When we do this we find $C_{T}^{SL}\approx 0.74$. This single estimate is somewhat lower than the first-principles mean estimate of 0.07, but it is clearly within the first-principle range 0.04–0.13.

Karyotakis et al (2015) has also experimentally measured the local outflow facilities for *in vivo* human eyes over the pressure range 20 mm Hg to 40 mm Hg. Nineteen subjects involved in this study were tested prior to cataract surgery, and had average age of 71.2 ± 4.1 years. We can estimate the value from *α* for this data by matching the ratios of the experimentally determined local outflow facilities with the local outflow facility ratios predicted by the model. When we do this, we find a good fit when *α* equals 0.069. The best statistical fit exponential curve to the experimental data was found by the authors to have a decay constant of 0.069 (with $C_{T}^{SL}$ somewhere between 1.0 and 1.05). Taking into account reported standard error of the mean for the mean local outflow facilities, the ±1 SEM range for *α* conceivably lies in the range 0.05 and 0.075. The first-principles mean value is 0.07 (range 0.04–0.013) versus the experimentally measured mean estimate 0.07 (range 0.05–0.075), it is apparent that the mean estimates agree while the estimated ranges completely overlap.

### Examples of estimates of outflow parameters based on pressure-volume and pressure-time curves for *in vivo* human eyes

We are now in a position to begin our estimation of outflow facility for the *in vivo* human eye. For manometric testing of the *in vivo* human eye, outflow facility estimation is usually estimated in two stages: (i) a pressure-volume curve is obtained as excess fluid is loaded into the eye, and (ii) a pressure-time test is recorded as this excess fluid leaves the eye (usually for 4 minutes).

First consider stage one of the manometric measurement—measurement of the pressure-volume curve and estimation of the ocular rigidity for the *in vivo* eye. Pallikaris et al (2005) reported tests on 79 subjects immediately prior to cataract surgery (mean age 65 ± 13.5 years, range 27 to 91 years) [69]. By directly injecting a known amount of fluid into the eye while measuring IOP, they obtained incremental pressure-volume curves for *in vivo* human eyes. From this data they estimated the mean ocular rigidity (*K*) for the group over the IOP range 10 mm Hg to 35 mm Hg to be 0.0290 (per microliter), with a 95% confidence interval on the mean estimate to be in the range 0.0257 to 0.0343 per microlitre [69]. For this group, they proposed that the volume-pressure data in living eyes could be approximated using straight-lines. Supporting this contention, they show the pressure-volume data for one patient (Fig 2 in Pallikaris et al (2005)). The data shown in this figure displays a near linear relationship between intraocular pressure (mm Hg) and volume of fluid injected into the eye (in microlitres) over the range pressure 0 mm Hg to 55 mm Hg. It is clear from equation(43) that such a linear relationship can only occur when *c*_0_ equals zero (see S1 Supporting Information Ocular Rigidity for definitions of *c*_0_ and *c*_1_). When *c*_0_ is zero the theoretical pressure-volume relationship is linear, and the ocular rigidity is inversely proportional to the ocular pressure. That is equation (43) reveals,

$$K=\frac{1}{c_{1}p}$$ (29)

It is important to realize that Eq (29) means the ocular rigidity is not constant, as often assumed in clinical analysis of eye measurements. It is particularly interesting to observe that for the data shown in Fig 2 in Pallikaris et al (2005), *c*_1_ is positive and equal to 1.43. This means that the ocular rigidity decreases with increasing intraocular pressure, which agrees with the earlier findings of [70, 71]. This decrease in ocular rigidity can be very substantial. To illustrate this for the data shown in Fig 2 of Pallikaris et al (2005), at 10 mm Hg the ocular rigidity is 0.0687, at 15 mm Hg it is 0.0458, further reducing to 0.0311 at 22.5 mm Hg and again reducing to 0.0172 at 40 mm Hg. In other words, for this individual the ocular rigidity at 40 mm Hg is only 25% of what it is at 10 mm Hg.

We note that for the data shown in Fig 2 of Pallikaris et al (2005), *c*_1_ is approximately six times the magnitude of the average *c*_1_ estimated by Silver and Geyer (2000). This attests to the large coefficient of variation observed for most eye parameters. We also note here that in a later study of 50 patients (20 men and 30 women) also undergoing cataract surgery, mean age 62 ± 12 years, over the IOP in range 15 mm Hg to 40 mm Hg, Dastiridou et al (2009) report that:

….pressure-volume relation in the human eye is generally in accordance with the results presented in Silver and Geyer (2000)….[and] The values for the coefficient of ocular rigidity are also similar to the results by Pallikaris et al (2005), but lower…the corresponding Friedenwald coefficient being 0.0098 [72].

We note here the mean ocular rigidity reported by Dastiridou et al (2009) is 0.0224 ± 0.005 at 37.5 mm Hg, while the mean ocular rigidity reported by Pallikaris et al (2005) (corrected from the Friedenwald coefficient reported by Pallikaris et al (2005)—see S1 Supporting Information Ocular Rigidity) is 0.0254 at 27.5 mm Hg.

Rather than being constant, the *in vivo* ocular rigidity is often a strong function of pressure, which has important implications for the estimation of outflow facility via pressure-time curves. For having a pressure dependent ocular rigidity changes both the pressure-time response of the eye (from which the outflow facility is estimated), and it changes the value of the ocular rigidity employed to estimate the outflow facility (see Eq (23)). For example, if *c*_1_ is strongly positive while the ocular rigidity is assumed to be constant over the pressure range of interest, then the outflow facility is substantially under-estimated at the higher end of the pressure range, and substantially over-estimated at the lower end of the pressure range.

Now consider ‘stage two’ of the manometric measurement—measurement of the pressure-time curve and estimation of the outflow facility for the *in vivo* eye, as described by [48]. Again prior to cataract surgery, Karyotakis et al (2015) estimates outflow facility based on manometric testing performed on 19 subjects (average age 71.2 ± 4.1 years).

The time dependent ‘pressure decay curve’ for a single patient is shown in Fig 1 of Karyotakis et al (2015). A volume-pressure curve was published by the same research group in 2013 (see Fig 1 of Detorakis et al (2013)), but it is clear this volume-pressure curve is not linear over the range 10 mm Hg to 40 mm Hg. Using equation (43) (see S1 Supporting Information Ocular Rigidity), the pressure-volume data shown in Fig 1 of Detorakis et al (2013) can be reasonably approximated with *c*_0_ = 45 and *c*_1_ = 0.2. We note that we have chosen fitting parameters that results in the ocular rigidity decreasing with increasing pressure, rather than the ocular rigidity increasing with increasing pressure (see S1 Supporting Information Ocular Rigidity).

**Example 1:** For this initial example, we use the pressure-time curve shown in Fig 1 of Karyotakis et al (2015), and the ocular rigidity obtained from the volume-pressure curve shown in Fig 1 of Detorakis et al (2013) and assume *p*_*T*_ equals 3 mm Hg. Using these two data curves, arbitrarily brought together from two different patients, we estimate the outflow facility by solving Eq (23) and so the total pressure dependent outflow.

A reasonable fit to the data is obtained (a highly accurate fit is not required for the purposes of illustration in this example) when *c*_0_ = 45, *c*_1_ = 0.2, *α* = 0.06 and ${\bar{C}}_{3}^{p}=C_{T}^{SL}{\bar{M}}_{3}^{p}=2.05{\bar{M}}_{3}^{p}$ microlitres/minute/mm Hg. Our estimated total pressure dependent outflow at 15 mm Hg is ${\bar{C}}_{3}^{15}(p_{15}-p_{3})=1.22(15-3)=14.6$ microliters/min. This is our first estimate of the total pressure dependent outflow at 15 mm Hg for the *in vivo* human eye.

$C_{T}^{SL}$ is high for this (arbitrary) data combination, as is the incremental volume change between 15 mm Hg and 40 mm Hg at 49.1 microlitres. However *α* is in the range estimated using first-principles (0.04 to 0.13). This example again serves to illustrate that the ocular rigidity has a profound impact on the estimation of model outflow parameters and so on the predicted outflow facility and total pressure dependent outflow. With this in mind, we note that Karyotakis et al (2015) also reported the mean constant ocular rigidity for the group to be *c*_0_ = 35.3 and *c*_1_ = 0 (which is quite different to the ocular rigidity parameters used in the first example). Karyotakis et al’s (2015) ocular rigidity constant was obtained from the pressure-volume curves of the nineteen subjects with an average age of 71.2 ± 4.1 years.

Analysing the pressure-time data using the experimentally measured ocular rigidity, the authors found the estimated group mean local outflow facilities are *C*^40^ = 0.0672 and *C*^20^ = 0.2652. Yet the analysis shown in Example 1 predicts *C*^40^ = 0.1917 and *C*^20^ = 0.6177. This very substantial discrepancy in the local outflow facilities clearly demonstrates that our initial Example 1 model, which brought together an arbitrary pressure-volume curve with an arbitrary pressure-time curve from two patients from two different groups, proves unsatisfactory when attempting to explain the Karyotakis et al (2015) findings. Clearly our model needs to use the mean ocular rigidity and the measured local pressure dependent outflow facilities reported by Karyotakis et al (2015) (though of course we may expect a better estimate using the patient specific pressure-volume and pressure-time curves, rather than using group mean values). This example illustrates the important point that simply choosing an arbitrary ocular rigidity results in unsatisfactory outflow facility estimates.

**Example 2:** The previous example suggests that if we want to estimate the outflow facility for a subject group, it is preferable to estimate each individual’s outflow facility and then take the mean, rather than first estimate model parameter means (e.g. ocular rigidity) and use these to estimate the mean outflow facility. But the second approach is what we have, and so we proceed with the data as reported by Karyotakis et al (2015).

If we now let *c*_0_ = 35.3 and *c*_1_ = 0 (reported mean value for group ocular rigidity), we can find a very good fit to the mean local outflow facilities reported by Karyotakis et al (2015). After parameter fitting the model, we find *C*^40^ = 0.067 is predicted by our model versus *C*^40^ = 0.0672 measured, and we find *C*^20^ = 0.266 is predicted by our model versus *C*^20^ = 0.2652 measured. From the ratio of *C*^20^/*C*^40^ we estimate *α* = 0.069. Then adjusting $C_{T}^{SL}$ to fit *C*^40^ = 0.0672 and *C*^20^ = 0.2652 while solving Eq (23), we find ${\bar{C}}_{3}^{p}=C_{T}^{SL}{\bar{M}}_{3}^{p}\approx 1.058{\bar{M}}_{3}^{p}$. We mention that taking into account additional *C*^*p*^ data reported between 20 mm Hg and 40 mm Hg does result in a lower estimate for $C_{T}^{SL}\approx 1.0$, which is closer the reported value by Karyotakis et al (2015).

For $C_{T}^{SL}\approx 1.0$ and *p*_*T*_ equals 3 mm Hg, we predict that total pressure dependent outflow at 15 mm Hg is $C_{3}^{15}(p_{15}-p_{3})=0.55(15-3)=6.6$ microliters/min. This is our second estimate of the total pressure dependent outflow for the *in vivo* human eye. This estimate is clearly the most reliable estimate of the two example estimates by far, as it is for a group of nineteen subjects with average age 71.2 ± 4.1 years, and the estimate is based upon experimentally measured mean local (or point) estimates of outflow facility at two different pressures.

We also mention that the mean pressure-time curve obtained for this reported data is somewhat higher compared to the individual’s pressure-time curve shown in Fig 1 of Karyotakis et al 2015, falling from 40 mm Hg to only 26.7 mm Hg within four minutes using the abovementioned mean parameters, rather than falling from 42.5 to 22.5 mm Hg within four minutes as shown for the individual pressure-time shown in Fig 1 of Karyotakis et al (2015). But such a difference is to be expected.

The estimated *α* = 0.07 is the same as our median ‘first-principles’ estimate of *α* = 0.07. Nevertheless the direct estimates in this second example are based on experimental measurements for *in vivo* human eyes, which gives us much greater confidence in this estimate than our initial estimates of *α* based on first principles using data assembled from a diverse variety of sources (though weighted towards reliable human data).

**Example 3:** Dastiridou et al (2013) reports the local outflow facilities at two pressures (25 mm Hg and 35 mm Hg) for two groups—control and glaucoma groups. In this example we consider the glaucoma group only (the next example we consider the normal control group). All eyes diseases were excluded other than cataract and glaucoma. There are 21 subjects in the glaucoma group (twelve with primary open angle glaucoma (POAG) and nine with pseudo-exfoliative open angle glaucoma (OAG)). The average age of the cataract patients is 75.4 ± 7.8 years, and 10 are male and 11 female—all were on medication for glaucoma treatment. Pre-operative mean IOP was 16 mm ± 5.0 mm Hg, so IOP is well-controlled by medication. Eye testing was performed immediately prior to cataract surgery.

Using the same method as described for the Karyotakis et al (2015) data, for this group of subjects the authors estimated experimentally that the ocular rigidity is *c*_0_ = 45.5 and *c*_1_ = 0, and *C*^25^ = 0.178 (±0.133) and *C*^35^ = 0.092 (±0.082). From the ratio of *C*^25^/*C*^35^ we find again *α* ≈ 0.069. Then we find ${\bar{C}}_{3}^{p}=C_{T}^{SL}{\bar{M}}_{3}^{p}\approx 1.0{\bar{M}}_{3}^{p}$. From this estimate, we predict that mean total pressure dependent outflow at 15 mm Hg is $C_{3}^{15}(p_{15}-p_{3})=0.55(15-3)=6.6$ microliters/min.

**Example 4:** Dastiridou et al (2013) reports the local outflow facilities at two pressures (25 mm Hg and 35 mm Hg) for two groups—controls and glaucoma groups. In this example we consider the control group only. All eye diseases other than cataract are excluded. There are 21 subjects in the control group. The average age of the cataract patients is 73.2 ± 5.5 years, and 12 are female and 9 male—none were on medications for glaucoma treatment. Pre-operative mean IOP was 14.5 mm ± 4.8 mm Hg.

Using the same experimental method as described by Karyotakis et al (2015), for this group the authors experimentally estimated that the ocular rigidity is *c*_0_ = 45.5 and *c*_1_ = 0 and *C*^25^ = 0.292 (±0.166) and *C*^35^ = 0.149 ((±0.085). From the ratio of *C*^25^/*C*^35^, which is very nearly identical to that found in Example 3, we find again *α* ≈ 0.069. We then scale the local outflow facilities to find ${\bar{C}}_{3}^{p}=C_{T}^{SL}{\bar{M}}_{3}^{p}\approx 1.65{\bar{M}}_{3}^{p}$. From this estimate, we predict that total pressure dependent outflow at 15 mm Hg is $C_{3}^{15}(p_{15}-p_{3})=0.90(15-3)=10.9$ microliters/min.

One interesting and potentially important observation here is that *α* ≈ 0.07 for both normal and well-controlled glaucoma groups is the same, while the total hydraulic conductance for the control group Dastiridou et al (2013) data is considerably increased (70%) relative to the glaucoma group. We also mention that the pressure-time curve obtained for this reported data is now similar to that shown in Fig 1 of Karyotakis et al 2015, falling from 40 mm Hg to 21.9 mm Hg within four minutes using these parameters (the pressure-time curve shown in Fig 1 of Karyotakis et al 2015 falls from 42.5 to 22.5 mm Hg in four minutes).

**Example 5:** One final, reasonably probable estimate of outflow facility parameters based on published data that may potentially be useful, is to pair the mean ocular rigidity reported by Silver and Geyer (2000) (*c*_0_ = 30.2, *c*_1_ = 0.242), with the individual pressure-time curve found in Fig 1 of Karyotakis et al (2015). When we do this, we find a good fit to the pressure time curve when *α* = 0.06, ${\bar{C}}_{3}^{p}=C_{T}^{SL}{\bar{M}}_{3}^{p}=1.5{\bar{M}}_{3}^{p}$. For this fit we find that at the local outflow facilities at 40 mm Hg is *C*^40^ = 0.14 (compare to Karyotakis et al (2015) data mean *C*^40^ = 0.0672) and at 20 mm Hg is *C*^20^ = 0.45 (compare to Karyotakis et al (2015) data mean *C*^20^ = 0.2652).

From these estimates, we predict that total pressure dependent outflow at 15 mm Hg is ${\bar{C}}_{3}^{15}(p_{15}-p_{3})=0.89(15-3)=10.7$ microliters/min. This is a higher estimate than Example 3. We note that the estimated *α* = 0.06 is now just below our estimated first-principles mean estimate, and well within range of *α* being between 0.04 and 0.013. This is our third and final estimate of the total pressure dependent outflow for the normal *in vivo* normotensive eyes.

### Summary of model parameter estimates

For our proposed model of outflow there are three key parameters controlling pressure dependent outflow: (i) the hydraulic conductance for the whole eye, $C_{T}^{SL}$ (microliters/min/mm Hg), (ii) the exponential decay constant, *α* (mm Hg)^-1^ and (iii), and no flow reference intraocular pressure *p*_*T*_ (mm Hg). We have employed animal and human *in vivo* data to make ‘first estimates’ of the parameters $C_{T}^{SL}$, *α* and *p*_*T*_. We have fitted our model to published data, and in so doing investigated the likely range of parameters controlling pressure dependent outflow. All the findings are summarized in Table 2.

**Table 2:** Estimates of model parameters (estimated range in brackets), and total pressure dependent outflow at 15 mm Hg.

Data Source		$C_{T}^{SL}$ (μliters/min/mm Hg)	*α* (mm Hg)^-1^	*p*_*T*_ (mm Hg)	Outflow at 15 mm Hg IOP (microliters/min)
First-principles estimate	SN	-	0.07 (0.04–0.13)^¶^	3 (2.5–3.9)	-
Barany (1964) (n = 45)	AN	1.0 (0.9–1.1)*	0.05	-	7.7
Brubaker (1975) (n = 10)	E	0.57	0.029 (0.023–0.035)*	2 (0–3.0)	5.8
Langham and Eisenlohr (1963) (n = 1)	N	0.74	0.05	-	5.8
Karyotakis et al (2015) (n = 19)	N	1.0–1.05	0.07 (0.05–0.075)*	-	6.6
Dastiridou et al (2013) (n = 21)	G	1.0–1.05	0.07	-	6.6
Dastiridou et al (2013) (n = 21)	N	1.65	0.07	-	10.9
Example 5	SN	1.5	0.06	-	10.7

N = normal *in vivo* human eyes, SN = estimate from data synthesized to approximate normal *in vivo* human eyes, AN = *in vivo* Vervet monkey eyes, E = enucleated human eyes, G = *in vivo* human eyes diagnosed with glaucoma but IOP well-controlled by medication (approximately 60% POAG and 40% pseudo-exfoliative OAG). Mean estimates for groups display considerable variability. It is certain that subject to subject variability is considerably larger.

* ±1 SEM

^¶^ estimated ±1 SEM

### Estimating the consolidation coefficient for the in vivo human eye

The consolidation coefficient *C*_*C*_ is defined by Eq (24), viz,

$$C_{C}=\frac{\hat{K}{\bar{C}}_{nt}^{p}p_{nt}}{V_{e-nt}}$$ (30)

Assuming a normal resting *in vivo* eye at 15 mm Hg, ranging from young adult to aged, plausible values for the ocular rigidity ($\hat{K}/V_{e-nt}$) are the range 0.01 to 0.03, and ${\bar{C}}_{nt}^{p}$ for reasonably small increments of IOP, is in the range 0.2 to 0.3. Then for normal eyes we estimate *C*_*C*_ (min^-1^) at 15 mm Hg as defined above to be in the range 0.03 to 0.14. It appears the most likely values are $\hat{K}/V_{e-nt}$ equals 0.028, ${\bar{C}}_{nt}^{p}$ equals 0.25 and *p*_*nt*_ equals 15 mm Hg, giving a most likely estimate of *C*_*C*_ equals 0.105.

## Discussion

### Implications for clinical practice and research

The interplay between net fluid production via active ion transport and pressure dependent outflow determines intraocular pressure. Because fluid outflow from the *in vivo* eye is pressure dependent, this complicates measurement and prediction of outflow facility [49], and the estimation of total pressure dependent outflow. Ideally a clinician would like to know the total pressure dependent outflow at every IOP for an *in vivo* eye (e.g. **Fig 1**). For in possession of such information, the local outflow facility, average incremental outflow facilities for any range of IOPs, and model parameters can be estimated, and so an assessment can be made of any deviation in model parameters from normal, which may assist diagnosis, prognosis or monitoring of eye disease. But in practice, we do not know how the total pressure dependent outflow changes with intraocular pressure.

Typically a clinician probes the eye system response by first inducing an incremental volume change, and then measuring the time dependent pressure response [33]. Based on this data, an ‘outflow facility coefficient’, *C* can be calculated using Grant’s equation (see Eq 2 [33]). The *C* found from Grant’s equation is usually *not* symbolically distinguished from the ‘conventional outflow facility’, also denoted *C*, which is defined using the modified Goldman equation (see Eq 1 [33]).

Goldman’s equation says the total pressure dependent outflow is the product of the outflow facility *C* and the ‘driving pressure’, where the driving pressure is defined as the difference in intraocular pressure and episcleral pressure. The outflow facility *C* in Goldman’s equation is usually interpreted as being primarily a ‘material property’ of the membrane and/or tissues through which fluid flows [43]. We notice that Grant’s equation assumes that the episcleral pressure changes a small amount during tonography [33], but this correction does not depend on the change in intraocular pressure. So with a fixed episcleral pressure, any change in driving pressure associated with elevated intraocular pressure is assumed to be solely due to the change in intraocular pressure. In contrast, the theory developed here assumes the pressure dependent outflow is primarily due to pressure dependent changes in the driving pressure (*p* − *p*_*ref*_).

It is reported that a surprisingly high fraction of outflow resistance (up to more than one half at normal intraocular pressure) resides distal to the trabecular meshwork [65, 73–76]. It is known there can be significant variation in resistance and variation in the diameter of episcleral veins [65, 76]. It is known there is a significant pressure gradient and pressure drop between the episcleral veins at the limbus and more distant ophthalmic veins (e.g. orbital veins, branches of the facial vein) [25, 63]. There may be significant resistance (and attendant pressure decrease) as aqueous humor flows through the network of aqueous filled vessels, which at some are point join blood filled vessels, which then join the episcleral vessels, which eventually join larger ophthalmic veins. Reports [75]:

After flowing through the trabecular meshwork, aqueous humor (AH) enters Schlemm's canal (SC), which expresses both blood and lymphatic markers; AH then passes into collector channel entrances (CCE) along the SC external well. From the CCE, AH enters a deep scleral plexus (DSP) of vessels that typically run parallel to SC. From the DSP, intrascleral collector vessels run radially to the scleral surface to connect with AH containing vessels called aqueous veins to discharge AH to blood-containing episcleral veins.

Exactly where we define our ‘membrane reference pressure’ for the conventional pressure dependent aqueous pathway is uncertain, but it is located somewhere between Schlemm’s canal and the larger ophthalmic vessels (the precise location at least partly depending on the duration of the experiment, the extent of eye tissues involved in distortions resulting from changes in IOP, and other eye state specific details). We also note there is significant non-linear variation in the driving pressure between the vitreous and the choroid interstitium over the pressure range 5 mm Hg to 40 mm Hg, as measured by direct cannulation of the choroid [23].

In both these cases, and indeed for all anatomical structures in the eye across which fluid flows, it is certain that increasing intraocular pressure distorts the local aqueous, lymphatic and blood networks in tissues of the eye through to the outside surface of the sclera, increasing aqueous and vascular outflow resistance [77–81], and thereby increasing fluid back-pressure in these flow networks. The degree of vascular distortion induced by the tissue loading from the IOP is to some extent mitigated by the fluid back-pressure in the aqueous, vascular and lymphatic networks, which mechanically holds the network vessels open against the tissue loading due the IOP that tends to collapse the vessels. The aqueous back-pressure also depends on both the magnitude and relative amounts of aqueous flow and blood flow through the aqueous and vascular networks, so there is a dependence on mean arterial pressure and orbital venous pressure [63]. The consequences of aqueous and vascular network distortion is also mitigated by autoregulation of the vascular network (which includes neural [76], autocrine and paracrine mechanisms), in ways that are only beginning to be understood [65]. For example increased back-pressure proximally may be offset by less back-pressure more distally, or vice-versa.

This increased aqueous and vascular back-pressure in turn increases local interstitial tissue pressures (interstitial fluid pressures are generally larger than aqueous or vascular pressures to ensure flow towards these networks), and this interstitial fluid pressure helps support the IOP. Because tissue is a saturated porous material, the tissue distortion of the extracellular matrix is controlled by the ‘solid stress’ or (‘effective stress’) in the tissue (which is equal to the total stress plus the interstitial fluid pressure). For eye tissues, the applied tissue loading is the IOP (which is equal to the applied ‘total stress’ on the inner surfaces of eye tissues i.e. solid stresses are zero at the inner surfaces of the eye). Tissue distortions are caused by drag forces on the solids in the tissue as aqueous fluid flows through the tissue. The key to understanding this system is to realise that a constant hydrostatic pressure causes no tissue distortion, while the tissue drag forces that cause tissue distortion are proportional to the fluid outflow rate. If drag forces are too large, then tissue distortion increases, which collapses aqueous and vascular networks and outflow reduces, and so drag forces reduce. On the other hand, if drag forces are too small, then tissue distortion is small and aqueous and vascular networks open up and outflow increases, and so drag forces increase. These two opposing processes reach an equilibrium state. It appears plausible that as IOP increases, the two processes involving the aqueous and vascular networks within the saturated porous media interact and reach a ‘limit equilibrium state’, in which the ‘driving pressure’ across the tissue becomes independent of IOP.

In the choroid and supraciliary space, the interstitial protein concentration (particularly albumin) may also influence the local interstitial fluid pressure and indeed fluid transport across the eye tissue or anatomical structure of interest. This is not included in the current model, and so this represents a model limitation. However we note that a constant osmotic suction has the effect of changing the no flow intraocular pressure *p*_*T*_ while leaving *α* unchanged). We also note that choroidal albumin concentration in the cynomolgus monkey is measured at between 2% and 12% of blood concentration [82]. While the smaller value for albumin concentration has negligible effect on the analysis presented here, the larger value has the effect of decreasing *p*_*T*_ by about 2.5 mm Hg.

Aqueous and vascular back-pressures and accompanying local increases in interstitial tissue pressures reduce local fluid driving pressures below what they would have otherwise been, and so pressure dependent outflows from the eye are less than what they otherwise would have been. Given this, it seems reasonable to suppose that the fluid driving pressure (*p* − *p*_*ref*_) changes due to eye tissue distortions as IOP increases, and so it appears reasonable to include this phenomena in a model of pressure dependent outflow from the eye.

At least for relatively short-term experiments, tissue distortions and attendant back-pressures induced by changing IOP are reversible, meaning the eye moves through ‘eye states’ that are reversible. So for example, these eye states are essentially the same for increasing IOP as for decreasing IOP [23, 43, 78]. Borrowing terminology from thermodynamics, the existence of a state function means that a particular eye state is independent of the path taken from an initial state to a final state. This suggests the existence of a ‘state function’ for outflow from the eye, so that eye states can be defined by state model parameters (e.g. the eye states can be defined by the theoretical model parameters such as *α*, $C_{T}^{SL}$, *p*_*T*_, IOP and net fluid production, as described herein).

The concept of a state function is reinforced by the observation that even for complete inversion (i.e. head-down position) causing substantial changes in venous back-pressure [66], at least in the short term, pressure dependent outflow from the eye, governed by *α*, $C_{T}^{SL}$, *p*_*T*_ and IOP, and the eye response continues to behave predictably. Postural inversion involves all outflow structures of the eye, not just the conventional outflow pathway, so the existence of a state function for the whole eye implies that all outflow pathways in the eye behave predictably. By this reasoning, the theoretical model proposed here may represent an ‘equation of state’ for the eye, but further testing of the model with experimental data is required to confirm this hypothesis.

There is no a priori reason to expect that the eye would behave as if there were a state function describing its behaviour, other than the existence of multiple, powerful homeostatic feedback processes (both biological and biophysical) at all levels of eye (and cardiovascular system). But each eye is different, and assuming state functions exist, every eye almost certainly has its own state function (i.e. each eye has its own model parameters).

In this paper we have developed a novel model for interpreting pressure-volume and pressure-time data, and so estimating incremental pressure dependent outflow and incremental outflow facility that takes account of ‘distortional pressures’ on eye outflow via changes in fluid driving pressure, (*p* − *p*_*ref*_). Like the Goldman equation, the pressure dependent outflow in this new model is the product of the outflow hydraulic conductivity and the driving pressure (*p* − *p*_*ref*_). We note that *C* in the Goldman equation applied to the conventional pathway is equivalent to $C_{con}^{SL}$ in the model presented here (the subscript *con* denotes conventional pathway). A non-linear pressure dependent driving pressure versus IOP is incorporated in the definition of outflow facility, so the outflow facility itself becomes pressure dependent while the hydraulic conductivity is assumed to be constant.

We note in passing that the pressure dependence of the outflow facility was noticed in the 1960s but attributed to changes in aqueous production rather than driving pressure for the pressure dependent outflow. For example, [41] comments: ‘Steady-state perfusion studies were too few to yield constructive information on this aspect, but it was of interest that, in both living eyes studied, the pressure-flow relationship was curvilinear and consistent with a significant suppression of aqueous humor formation by an increase in intraocular pressure’.

But numerous experimental studies have shown the outflow facility is pressure dependent (including flow at the ciliary body, which makes a minor contribution to total pressure dependent outflow), so we need to denote the average outflow facility using the new notation ${\bar{C}}_{p_{1}}^{p_{2}}$, which precisely defines the pressure range over which an average outflow facility is measured or calculated. For the purposes of the theory developed here $C_{T}^{SL}$ is taken to be constant, which is probably a good approximation for the short term tests employed to measure outflow facility. However $C_{T}^{SL}$ is generally time dependent, and the model can accommodate a pressure dependent $C_{T}^{SL}$ if data suggests a need to explicitly include this (i.e. via *α*_2*i*_ as described in the theoretical development).

For the new model presented here, three key parameters govern pressure-dependent outflow, namely: (i) the hydraulic outflow conductance for the whole eye (denoted $C_{T}^{SL}$), (ii) the exponential ‘decay constant’ describing the rate of decrease of local outflow facility with IOP for the whole eye (denoted *α*) and, (iii) the IOP at which there is zero pressure dependent flow into or out of the whole eye (denoted *p*_*T*_). Using the theory presented here, if these three pieces of information are known the total pressure dependent outflow at every IOP for an *in vivo* eye is known, and so all pressure dependent outflow facilities can be estimated, including those measured in the clinic (i.e. this is typically ${\bar{C}}_{nt}^{p_{2}}$, where *p*_*nt*_ is the patient specific normotensive pressure).

We assume that a clinician wanting to implement this theory will have available two estimates of incremental outflows at two different IOPs, or two estimates of average outflow facilities over two pressure ranges (i.e. they have two points on a curve shown in **Fig 5**, obtained from pneumatonography at two different weights). The ‘thorny problem’ that presents itself is how to reconstruct the total pressure dependent outflow curve (or equivalently, estimate the three model parameters) from two estimates of incremental outflows or average outflow facilities. Unfortunately this is not possible for one key reason: while we can estimate both $C_{T}^{SL}$ and *α* assuming *p*_*T*_, in fact we do not know and cannot estimate *p*_*T*_ measuring incremental outflows (e.g. even three measurements of incremental outflows cannot fix *p*_*T*_, though it can reduce uncertainty in the estimates of $C_{T}^{SL}$ and *α*). To estimate *p*_*T*_ we need to know either at least one total outflow at one IOP, or one driving pressure for the whole eye at one IOP.

In a research context, it may be possible in a small group of patients to observe the rate of subretinal fluid drainage following retinal detachment [18], and also measure aqueous outflow fluorometrically [83], so enabling an estimate to be made of total outflow. Another potential way to try and circumvent this problem in the clinic would be to estimate the outflow or driving pressure for the conventional outflow pathway [60, 83], and use estimated whole eye parameters $C_{T}^{SL}$ and *α* to then estimate the no-flow pressure *p*_*Tcon*_. *p*_*Tcon*_ would then be assumed to be representative for the whole eye. But in addition to being practically challenging, clearly this would again be subject to both modelling and measurement error, and requires further research to know if it is a clinically useful approach. Clearly a significant practical problem when estimating model parameters from the incremental outflows is measurement error.

In this manuscript we have attempted to managed these two problems by providing a pressure dependent outflow model that uses precisely defined parameters to interpret measured data (i.e. by precisely defining ${\bar{C}}_{p_{1}}^{p_{2}}$), and by using a conservative value for *p*_*T*_, meaning making a choice that results in a probable low estimate of total pressure dependent outflow from the eye. We have shown the estimate of *p*_*T*_ = 3 mm Hg is in reasonable agreement with a range of available human and animal data (see first-principles parameter estimation summarized in Table 2). The most convincing evidence available is for the conventional outflow pathway, where the total outflow at normotensive pressure has been extensively investigated and so known. It is demonstrated here that *p*_*Tcon*_ is reasonably estimated to be about 3 mm Hg. While this is encouraging progress, the reliability of the estimated *p*_*T*_ estimate for the whole eye remains somewhat uncertain, and clearly further research is required.

However we can estimate *α* for the whole eye by first taking the ratio of the incremental outflows at the known IOPs, and finding the curve in **Fig 4** that has the same ratio of incremental total outflows at the two known IOPs. Once *α* is known, we can then scale $C_{T}^{SL}$ from **Fig 4** (**Fig 4** is constructed for $C_{T}^{SL}=1$). We can scale from **Fig 4** because the ratio of the hydraulic conductivities (actual hydraulic conductivity to the one assumed in **Fig 4**) has the same proportion as the ratio of the pressure dependent outflows (i.e. the actual incremental outflow (at known IOP) to the one shown in **Fig 4 (**at known IOP and the now known *α*)). Of course the precision and accuracy of incremental outflow estimates depends on model uncertainty (including any assumptions about model parameters), and the accuracy of the estimate depends on the precision and accuracy of the measurements of average outflow facility.

This is probably where the clinical relevance of the model proposed in this paper rested, until [47] and [48] published what are effectively estimates of local outflow facilities (denoted here *C*^*p*^) for the aged *in vivo* human eye. As far as we are aware, this is the first published data on local outflow facilities for the *in vivo* human eye. In the context of our theory, these papers are important publications because they demonstrate that the local outflow facility for *in vivo* human eyes is reasonably approximated by an exponential decay function for the whole eye. This fact substantially reduces potential modelling uncertainty. Nevertheless our ‘thorny problem’ remains: how to reconstruct the total pressure dependent outflow curve from estimates of local outflow facilities.

The process of reconstructing incremental pressure dependent outflow curves from estimates of the local outflow facility is most simply found by evaluating a definite integral between the ocular pressures of interest (see Eqs (14) and (26)). The only real difficulty that presents itself when reconstructing the total pressure dependent outflow curves is again not knowing the IOP at which there is no pressure dependent flow into or out of the whole eye, that is *p*_*T*_. Typically we can estimate *α* by first taking the ratio of two measured local outflow facilities, and finding the curve in **Fig 3** that has the same ratio. Once *α* is known, we can then scale $C_{T}^{SL}$ so two (or more) estimated local outflow facilities have the same magnitude as the measured local outflow facilities. Once again, the precision and accuracy of outflow estimates again depends on model uncertainty, model assumptions, and on the precision and accuracy of the measurements of local outflow facility. But given the exponential decay curve fits the experimentally measured human *in vivo* local outflow facilities very well, model uncertainty for the *in vivo* human eye is considerably reduced.

Model uncertainty is further reduced by our reanalysis of the experimental data presented by [39] and Brubaker (1975). We have found the exponential model fits their data reasonably well too. The data presented by Brubaker (1975) is extraordinary in so far as the authors are aware, it is one of the few publications that presents the total pressure dependent outflow for the whole eye across nearly the entire range of IOPs from 10 mm Hg up to 50 mm Hg. While the data is for human eyes, the only drawback is that the data is obtained as an average of data from *enucleated* human eyes. Nevertheless in terms our model this data suggests the no-flow IOP (*p*_*T*_) is from zero up to 3.0 mm Hg. Interestingly we notice that a ‘critical closure pressure’ has been reported in enucleated rabbit eyes (where *p*_*T*_ is reported to be about 7 mm Hg [76]). Though additional explanations for a threshold pressure are possible *in vivo* (e.g. active cell contraction), the presence of a threshold flow pressure *p*_*T*_ in enucleated eyes is consistent with a residual compressive (solid) stress in the extracellular matrix, so that aqueous and vascular networks are shut by the residual compressive stress, and so there is no flow below the threshold IOP pressure *p*_*T*_.

And we again note that extrapolation of direct cannulation data for choroidal interstitium in cynomolgus monkeys also suggests that *p*_*T*_ around 3 mm Hg is a plausible *in vivo* estimate for this outflow pathway [23]. And in addition the two analyses of conventional pathways for *in vivo* in human eyes also suggests that *p*_*T*_ is around 3 mm Hg. So this data is consistent with our modelling assumption (*p*_*T*_ is 3 mm Hg) used throughout the calculations presented in the Results section (unless stated otherwise). However it is likely that this value changes with *in vivo* conditions and from eye to eye. It seems a plausible range for a normal *p*_*T*_ in the normal eye is probably in the range from minus a few mm Hg up to positive 4 or 5 mm Hg.

We now turn to consideration of the most likely estimate for the decay constant *α*, which in the theory formulated here is responsible for decreasing pressure dependent outflow facility with increasing intraocular pressure. Our ‘first-principles’ estimate for the decay constant is grounded in the reported changes in choroidal interstitial pressures for cynomolgus monkeys and the *in vivo* resting episcleral venous pressure in humans, but heavily weighted towards the more reliable human data. Based on this mixture of animal and human data, we estimated the median decay constant in normal young adult eyes to be about *α* = 0.07, with a normal range for *α* estimated to be 0.04–0.13.

Based on experimental data for the aged-adult and apparently normal *in vivo* human eyes, [47] and [48] calculate local outflow facilities and then statistically fit this data to an exponential function. The statistical fitting parameters turn out to be our $C_{T}^{SL}$ and *α*, which in our theory are physiological meaningful model parameters, rather than simply statistical curve fitting parameters. Karyotakis et al (2015) estimated the scale fitting constant to be 0.997, while the exponential decay constant is estimated as 0.069. We note that to estimate the local outflow facilities, Karyotakis et al (2015) assumed a constant ocular rigidity in their data analysis (and when we use the reported values, we also obtain similar estimates). Importantly, the data and curve fitting estimate for the group of 19 subjects (average age 71.2 ± 4.1 years) is clearly consistent with the normal range for the decay constant based on our independent first-principles estimate. Pleasingly the range for first-principles estimate for *α* (0.04 to 0.13) and the range for the experimentally measured *in vivo* estimate of *α* (0.05 to 0.075), completely overlap, giving greater confidence in both estimates for normal *in vivo* human eyes (see summary of results shown in Table 2).

While any difference in these estimates may be partly related to age of the subjects whose data was employed for these two estimates, and partly related to differences in the pressure range over which the estimates are made, clearly the experimentally measured estimate for the *in vivo* human eye by [47] and [48], are more reliable estimates than our first-principles estimate. The first-principles estimate suffers from inadequate data about flow across the retinal pigmented epithelium, and the necessary accompanying assumptions made along the way to obtain the first-principles estimate. For example, the use of data obtained from cynomolgus monkeys rather than humans, differences in pressure ranges, and uncertainty about the fractions of flow exiting the anterior and posterior chambers all make the first-principles estimate less reliable.

As discussed in the development, our theory is somewhat indefinite about the specific physical origin of the decay constant (e.g. IOP induced tissue distortions result in changes in interstitial fluid pressure, and aqueous, lymphatic and vascular network back-pressures), and so indefinite about the origin of the decreasing local pressure dependent outflow facility with increasing intraocular pressure. However based on the analysis of experimental published data shown above, it seems likely that for IOPs up to about 40 mm Hg for human subjects, the proposed theory appears to fit the existing experimental data rather well.

Nevertheless we also need to keep in mind that there are almost certain changes occurring over longer time scales, due to differential gene expression and long-term tissue remodelling, not to mention changes due to disease states. In this context, it is interesting to note the remarks by [75] about the location and mechanism by which (free) energy dissipation in the aqueous outflow occurs. These authors comment:

Distal resistance and its relationship to the [trabecular meshwork] TM and [Schlemm’s canal] SC are key considerations to define outflow resistance in glaucoma. We must determine whether distal resistance means distal to the TM or distal to SC external wall, which represent two distinct mechanisms. The distinction has ramifications for both theoretical constructs for outflow resistance and minimally invasive glaucoma surgical approaches. If distal indicates distal to the TM inner wall, then TM tissues still may have a central role in resistance, since TM tissues can change their location and configuration. Such resistance changes can occur, since the trabecular tissue distends into SC to occlude SC lumen, distends into the collector channels, compresses SC structures, and reconfigures the CCE at SC external wall through their connections.

If distal refers to only structures that are distal to the SC external wall, then distal resistance alternatives are limited to the CCE and the intrascleral collector channels in the deep scleral plexus (DSP), which course through the sclera to the episcleral veins and the aqueous veins. Identifying the TM, SC, and the distal outflow pathways as possible discrete sites of resistance raises the possibility that resistance occurs in series. This hypothesis suggests that resistance sites act synergistically with a spectrum of relative inputs that act in concert to synchronously regulate AH outflow and determine IOP.

Given the uncertainty about the precise location of (free) energy dissipation, it seems appropriate that our theory is somewhat indefinite about exactly where the reference pressure is measured, and indefinite about the multiple mechanisms and their interactions underlying the decay constant *α*. What is known is that the outflow facility is pressure dependent, and it is shown here that the observed pressure dependence of outflow facility is reasonably well explained using the theory described herein. So from one perspective, the theory itself can stand independent of any physical mechanism that attempts to explain it, and still be regarded as a useful theory if it makes useful predictions.

For example, the qualitative structure of estimated total pressure dependent outflows show in **Fig 1** is of considerable practical interest. The various curves show that when the decay constant *α* becomes sufficiently large, the total pressure dependent outflow curve flattens, and eventually becomes effectively constant. For example when *α* = 0.15, the total pressure dependent outflow is rather flat from an intraocular pressure of about 15 mm Hg and higher, and is almost completely flat above an intraocular pressure of 40 mm Hg. This is confirmed by looking at **Fig 3**, which shows the local outflow facility approaches zero as intraocular pressure increases above about 30 mm Hg. According to the theory developed here, this occurs physically because a unit increase in intraocular pressure is matched by a unit increase in membrane reference pressure, so when *α* = 0.15, over the range 15 mm Hg to 40 mm Hg there is very little net increase in driving pressure across eye membrane with increasing intraocular pressure.

These observations may provide an explanation for why some eyes exhibit large circadian variations in intraocular pressure. For when the total pressure dependent outflow is ‘flat’ with respect to IOP, comparatively small circadian variations in net eye fluid production [84], will result in large variations of IOP. The practical effect is that as *α* becomes large, intraocular eye pressure becomes unstable. Eyes with large and unstable variations in intraocular pressure are reported to exist. For example, Wilensky (1991) classifies diurnal variations in intraocular pressure as ‘flat’, ‘rhythmic’ and ‘erratic’, with the percentage of erratic diurnal variations increasing from 0% in normal eyes, to 14% in those diagnosed with ocular hypertension to 22% in those diagnosed with glaucoma. Indeed large diurnal intraocular pressure fluctuations have been identified as an independent risk factor for glaucoma progression [85, 86].

To give another example testifying to the model’s potential theoretical usefulness, we consider uveoscleral (unconventional) outflow. Tian et al (2006) explains:

In normal living monkeys, trabecular outflow is pressure sensitive and uveoscleral outflow is comparatively pressure-insensitive when IOP is greater than 7 to 10 mmHg. Although, under some circumstances, such as at low pressures…. uveoscleral outflow may not be completely pressure insensitive, pressure-insensitive outflow typically represents primarily uveoscleral outflow… Based on Bill’s studies when the pressure is elevated from P0 to P1 at the beginning of perfusion, uveoscleral outflow will increase pressure-dependently until the pressure reaches a specific point (e.g., 7–10 mmHg). However, when the pressure is elevated from P1 to P2 during perfusion, the uveoscleral outflow will be no longer pressure sensitive.

In terms of the present theory, we see that so-named ‘unconventional’ uveoscleral outflow pathway may in fact be quite ‘conventional’ in the sense that it can be regarded as pressure dependent outflow with an unusually large *α*. For example if *α*_*uv*_ = 0.25 (i.e. about 3 to 4 times larger than the normal *α* for the whole eye) then 92% of the maximum total outflow is attained at 10 mm Hg, while 98% of the maximum total outflow via this route is attained at 15 mm Hg. For all higher IOPs, for all practical purposes uveoscleral outflow can be regarded as pressure independent. We see the theory presented here in a small but useful way provides insight, creating a more complete ‘mental model’ of uveoscleral outflow, one that subtly changes our understanding of pressure dependent outflow from the whole eye.

Contemplating the theory presented here, many new hypotheses present themselves. One hypothesis is that eyes showing erratic or usually large circadian variations in IOP [87] are likely to have a large *α*. An alternate hypothesis is that hydraulic conductivity for the eye, $C_{T}^{SL}$, may decrease sufficiently for similarly unstable behaviour to occur. Yet another hypothesis might be that both *α* and $C_{T}^{SL}$ increase with age, with the beneficial increase in $C_{T}^{SL}$ normally offsetting the deleterious increase in *α*, which together maintain a normal IOP with increasing age. However if *α* increases more quickly than $C_{T}^{SL}$, then ocular hypertension and eventually glaucoma may result. To examine these hypotheses requires more extensive data sets to better define parameter distributions and establish any correlations between *p*_*T*_, *α* and $C_{T}^{SL}$, and how this may change with age.

Finally we reflect further on the analysis of the ‘inversion experiment’ reported in the Results. We found that immediately following inversion, the measured parameters were consistent with the model proposed here, and the experimental results were readily parameterized. But over an unspecified period of time, under the influence of the elevated hydrostatic head of about 22.5 mm Hg, in terms of the current model it seemed likely that the no-flow intraocular pressure increased from about 2.5 to 7.75 mm Hg, an increase of 5 mm Hg, and this change enabled aqueous production to return to its former rate.

But there is an alternative outflow model that takes into account an (additive) constant background pressure *p*_*B*_ (as mentioned in the development), which can be included by a simple modification of the theory that will be examined in a future paper. This modification includes the additional parameter *p*_*B*_ that enables the outflow curves to be translated horizontally (as we know, the parameter *p*_*T*_ enables the outflow curves to be translated vertically). In this model extension, the original model curves in the all the above figures can slide along the IOP axis under the influence of a background pressure. Experimental results consistent with this theory have been published [88]. Based on the theory of porous media (which says tissue deformations are not controlled by total stresses, but rather by effective or solid stresses, and hydrostatic pressure causes no tissue deformation), it appears this background pressure model is grounded with a sound theoretical basis.

This range of theoretical models appears to suggest that immediately after inversion, the first model is a good representation of what was observed experimentally. But then over a relatively short time (e.g. half an hour), the background pressure in the whole eye increases to a constant back-ground value, and the second model is then explanatory. To obtain the same rate of fluid production in the inversion experiment as prior to inversion, the background pressure *p*_*B*_ throughout the eye needs to be about 18 mm Hg, while *p*_*T*_ remains unchanged. In this case, we would expect eye pressure to return to normal upon assuming an upright position, once excess fluid in the eye had drained. Finally, the first model predicts that in the long-term, *p*_*T*_ increases by about 5 mm Hg (possibly due to tissue remodeling). If tissue remodelling is responsible for this change, then we would expect that it would take some days, weeks or months to achieve. And because tissue remodelling cannot be quickly reversed, we expect that IOP would not return to normal immediately upon resuming an up-right position.

We note that following surgical correction of spontaneous multiple A-V fistulas that were increasing venous pressure in one eye (which is physiologically analogous to a reversal of prolonged inversion), [89] reports: ‘At the second month of follow-up…the IOP elevation had regressed’, suggesting it takes some time for IOP to return to normal. It is yet unclear if this proposed sequence of events is correct, but the model predictions described here can be readily discriminated experimentally, as their respective responses to further incremental IOP increases are very different. However it is possible all the modelling predictions are correct, and indeed it is likely that the real eye behaves with elements of several models that are connected to each other through time-dependent model parameters. Clearly this requires further investigation.

### Estimates of pressure dependent outflow and outflow facilities for the eye

The experimental measurement of outflow facility for the *in vivo* eye is challenging. For *in vivo* intracameral measurements on human eyes, readings are confounded by breathing, intraocular pulsations and any eye movement. Estimating outflow facility accurately based on pressure-time curves is critically dependent upon the accurate estimation of ocular rigidity, as outflow facility estimates change significantly with microliter variations in the volume of excess fluid estimated to be draining from the eye. Ocular rigidity estimation relies upon accurate estimation of the pressure-volume curve for each individual eye. Even seemingly good coefficients of determination for the pressure-volume curve (e.g. a curve fit to the data with an r^2^ value of say 0.95) can lead to a significant error in the estimation of outflow facility compared to a curve fit with an r^2^ value of 0.99. For this reason the analysis presented here strongly suggests that using an adjustable pressure dependent ocular rigidity, such as the one developed by Silver and Geyer (2000), can result in significantly more accurate estimates of outflow facility relative to those estimates assuming a constant ocular rigidity.

Based on the data presented in (Karyotakis, Ginis et al. 2015), our analysis suggests that for a group of 19 cataract patients, average age 71.2 ± 4.1 years, with no known eye disease, the mean average outflow facility (${\bar{C}}_{3}^{15}$) is around 0.55 microlitres/minute/mm Hg. At normal intraocular pressure we estimate the total pressure dependent outflow to be 6.6 microlitres/minute (see Results, Example 2). This conservative estimate is more than twice as large as the reported average anterior chamber rates of aqueous formation (at around 2.4 to 3.0 microlitres/minute—normal range 1.8 to 4.3 microlitres/minute [21, 27, 28, 51, 64, 90].

However in this context we note that Karyotakis et al (2015) also reported *median* local resistances at several IOPs (local resistance is the inverse of local outflow facility), which are somewhat different to the mean local resistances. Fitting an exponential curve to the median local outflow facilities we find $C_{T}^{SL}$ is then about 0.85 microlitres/min/mm Hg, and estimated median pressure dependent outflow would then be about 5.6 microlitres/min. However in the following, we work with the mean pressure dependent outflow estimate rather than the median estimate, as mean estimates are most often reported in the literature.

These model estimates are broadly consistent with fluorometric measurements of total aqueous outflow measured at 5.2 ± 1.9 microlitres/minute for Beagle dogs [91]. The outflow facility for Vervet monkeys (n = 45) is reported by [39] to be around ${\bar{C}}_{12}^{21.4}$ equals 0.55 microlitres/minute/mm Hg, with a ‘resting intraocular pressure’ *p*_0_ of approximately 9.1 mm Hg. Our reanalysis of the data shown in Fig 7 of Barany (1964), indicates that *α* is approximately 0.05, rather than zero as suggested by [39]. We observe that the estimated *α* ≈ 0.05 is somewhat lower than the first-principles estimates for *α* (mean 0.07 and range 0.04 to 0.13), and somewhat lower than the estimate for aged human eyes (*α* around 0.07). The estimate for Vervet eyes of $C_{T}^{SL}\approx 1.0$ (range 0.90 to 1.10) appears reasonable when compared that for the *in vivo* aged human eyes ($C_{T}^{SL}\approx 0.74$, and $C_{T}^{SL}\approx 1.0-1.7$). Estimates of total pressure dependent outflow for Vervet monkey eyes *in vivo* is around 4.45 microlitres/min at 9.1 mm Hg (resting IOP), is consistent with the 5.2 microlitres/min *in vivo* estimate for Beagle dogs [91] and with the 6.6 microlitres/min we estimated above for aged *in vivo* human eyes.

Brubaker (1975) reports testing ten, enucleated human eyes less than 24 hours after enucleation, with ‘episcleral tissue and bulbar conjunctiva carefully removed’, using a technique that ‘minimises the artefactual effects of ocular stretching and anterior chamber deepening’ (no details of age are given). At 15 mm Hg measured mean total pressure dependent outflow averaged over the 10 eyes is around 5.85 microlitres/min (see Fig 2 and Table I [43]). If the average data shown in Fig 2 in Brubaker (1975) is fitted to an exponential decay curve, prediction accuracy is excellent when *p*_*T*_ equals 2 mm Hg, *α* ≈ 0.029 (range 0.023 to 0.035) and $C_{T}^{SL}=0.57$. This estimate for *α* is below our estimated range for *α* based on our first-principles estimate, which employed data from *in vivo* eyes (*α* range 0.04 to 0.10), but we note that when *p*_*T*_ equals 3 mm Hg, *α* ≈ 0.038 (range 0.030–0.045) and, which is at the lower end of our estimated range for the *in vivo* eye.

It appears that *p*_*T*_, *α* and $C_{T}^{SL}$ are all lower for enucleated eye tests relative to *in vivo* eye tests, which is perhaps not surprising given the substantive differences between *ex vivo* and *in vivo* eye conditions, even though the pressure dependent outflows at normotensive pressures are predicted to be similar. Brubaker (1975) pressure tested the eyes through an ‘up-down’ or ‘down-up’ pressure sequence twice, and we note in passing that *α* reduces significantly between first and second testing (from *α* equals 0.041 on the first testing to 0.024 on the second testing, at *p*_*T*_ equals 2 mm Hg and $C_{T}^{SL}=0.60$), which appears to be indicative of further significant changes occurring in the eye due to the test procedure itself. For example pressure dependent outflow at 50 mm Hg is 11.70 microlitres/min on the first test, but this increases to 16.55 microlitres/min on the second test (a 40% increase).

Nevertheless, the enucleated eye data presented also demonstrates that an exponential approximation of local outflow facility provides an accurate representation of pressure dependent outflow across the pressure range 10 mm Hg to 50 mm Hg. Further, it can be observed from Table 2 that for enucleated human eyes $C_{T}^{SL}\approx 0.57$ microliters/min/mm Hg when *p*_*T*_ equals 2 mm Hg, but this rises to $C_{T}^{SL}=0.70$ when *p*_*T*_ equals 3 mm Hg, which is consistent with other *in vivo* estimates of $C_{T}^{SL}$ shown in Table 2. For example the estimate of $C_{T}^{SL}\approx 0.70$ microliters/min/mm Hg is very close to the estimate based on *in vivo* data presented by [41] for a single subject (i.e. $C_{T}^{SL}\approx 0.75$ microliters/min/mm Hg and *α* ≈ 0.05).

Interestingly we can also employ the model to analyse the incremental outflow facility for the whole eye based on pneumatonometry and pneumatonography data reported in [33]. Fifty six eyes from 28 healthy participants were measured (age range 41 to 68 years). Using the standard assumed ocular rigidity constant, the measured outflow facility was 0.24 ± 0.08 microliters/min/mm Hg, but if the measured ocular rigidity for the group was used, the measured outflow facility was found to be 0.21 ± 0.07 microliters/min/mm Hg. If we assume that for *in vivo* human eyes $C_{T}^{SL}\approx 0.7-1.0$ microliters/min/mm Hg, *p*_*T*_ equals 3 mm Hg, and that the mean intraocular pressure for the test is about 28 mm Hg, then *α* can be read directly from **Fig 5** (or finding the incremental pressure dependent outflow from the incremental facilities, using **Fig 4** that is more accurately read). When $C_{T}^{SL}=1.0$ microliters/min/mm Hg, *α* is estimated to be about 0.075. When $C_{T}^{SL}=0.7$ microliters/min/mm Hg, *α* is estimated to be about 0.055. Pleasingly, we observe these estimates are close to $C_{T}^{SL}\approx 0.75$ microliters/min/mm Hg and *α* ≈ 0.05 estimated from the single subject reported in [41], while the 0.075 estimate is close to the first-principles estimate (0.07). Most importantly, this estimate is close to the model estimated *α* based on the data presented by [47] and [48] (see Table 2). Of course if the incremental outflow facility is measured over a second pressure range, both *α* and $C_{T}^{SL}$ can be fixed. In this context we note that more accurate mean estimates of *α* and $C_{T}^{SL}$ can be made if based on patient-specific data (rather than group averages), and again more accurate estimates of *α* can be made if patient-specific incremental and/or local outflow facilities (based on an accurate volume-pressure curve) are measured at a minimum of two IOPs.

By examining multiple small data sets currently available, we see that a plausible range for models parameters can be gradually established, but clearly a great deal of additional clinical and basic research needs to be done to establish the distributions of model parameters, to establish model parameter correlations and how these may change with age and in disease states. To help further the process of model parameter estimation for specific outflow pathways, we now attempt to draw together our insights based on all the previous analysis using the new model (and from the analysis of pseudofacility explained in the next section). Here we summarize our own ‘best estimates’ for model parameters that describe pressure dependent eye outflow from a normal eye at 15 mm Hg, namely: for pressure dependent outflow through anterior pathways $C_{con}^{SL}\approx 0.33-0.43$ microliters/min/ mm Hg (driving pressure 7 mm Hg), for retinal pigmented epithelium/choroidal pressure dependent outflow $C_{ch}^{SL}\approx 0.45-0.60$ (driving pressure 5 mm Hg), for pressure dependent outflow across the ciliary body (pseudofacility) $C_{cb}^{SL}\approx 0.06-0.11$ (driving pressure 11 mm Hg). These pathway specific estimates give an estimated pressure dependent outflow range from the whole eye from 5.3 to 7.3 microlitres/min. We note in passing that eye size is one factor contributing to these ranges, and with appropriate subject-specific data, it may be possible to reduce these ranges.

The mean hydraulic conductivity for the whole eye is then in the range $C_{T}^{SL}\approx 0.84-1.14$, and the mean driving pressure for outflow from the whole eye is around 6.4 mm Hg. *p*_*T*_ is probably normally in the range 0 to 3 mm Hg. Assuming *p*_*T*_ is 3 mm Hg and pressure dependent outflow is 6.6 microlitres/min, then the following model parameter values are probable: $C_{con}^{SL}\approx 0.40$, $C_{ch}^{SL}\approx 0.55$ and $C_{cb}^{SL}\approx 0.075$ suggesting $C_{T}^{SL}\approx 1.025$, and the mean driving pressure at 15 mm Hg is then 6.4 mm Hg for *α* equal to 0.0725 mm Hg^-1^. We estimate the average outflow facility ${\bar{C}}_{3}^{15}$ as 0.55 microlitres/min/mm Hg for the whole eye with *α* equal to 0.0725 mm Hg^-1^, and the component average outflow facilities are: for anterior flow pathways 0.215 microlitres/min/mm Hg, for retinal pigmented epithelium/choroidal pathway 0.295 microlitres/min/mm Hg and for the ciliary body flow pathway 0.04 microlitres/min/mm Hg (sum 0.55). But the component average outflow facilities based on actual driving pressures and/or outflows (or from pathway specific alphas) for each tissue or anatomical structure (rather than the average driving pressure or the average alpha) are: for anterior flow pathways 0.23 microlitres/min/mm Hg, for the retinal pigmented epithelium/choroidal pathway 0.23 microlitres/min/mm Hg and for the ciliary body flow pathway 0.07 microlitres/min/mm Hg (sum 0.53).

Finally assuming aqueous hypertonicity is 5 mOsm relative to its reference fluid (equivalent to about 100 mm Hg osmotic suction), then the driving pressure for inflow is (100–11) 89 mm Hg, and the inflow from the ciliary body is then 89 times 0.075, that is 6.7 microlitres/min, which approximately provides mass balance with the pressure dependent outflow from the whole eye (compare 6.6 microliters/min).

### Implications for eye physiology

Taken together, these estimates for total pressure dependent outflow do create a clear and significant discrepancy in estimates between the predicted *in vivo* pressure dependent outflow estimates (i.e. mean estimate of 6.6 microlitres/min—see Results, and Examples 2, 3 and 4 using [48] and [47] data, and the summary of Results shown in Table 2), and reported aqueous production rates by the ciliary body (i.e. 2.4 to 3.0 microlitres/min [21, 27, 28, 51, 64]).

We first wish to point out that we have at each stage employed modelling options and results that minimise our predicted *in vivo* pressure dependent outflow. For example the total pressure dependent outflow based on control group in the Dastiridou et al (2013) data predicts an much higher 10.9 microlitres/min (see Results section Example 4 using Dastiridou et al (2013) data, or summary Table 2). And when we pair the high quality pressure-volume data of Silver and Geyer (2000) with the calculated pressure-time curve for Karyotakis et al. 2015 (see Results section Example 5), we find the predicted *in vivo* total pressure dependent outflow is 10.7 microlitres/min. Again this is significantly larger than our mean estimate 6.6 microlitres/min. The estimates of 10.9 and 10.7 microlitres/min are clearly qualitatively correct, as a larger hydraulic conductance and a more compliant eye means that more fluid can be expelled from the orbit in the same period of time, resulting in estimates of total pressure dependent outflow higher than 6.6 microlitres/min, which we have chosen as a conservative estimate. Further based on a range of experimental data, we have also chosen our IOP reference pressure as 3 mm Hg rather than 2 mm Hg or perhaps zero mm Hg, which results in conservative estimates of total pressure dependent outflow. All this suggests the apparent discrepancy between estimated total pressure dependent outflow and aqueous production noted above may well be real, and so should be rationally explained in some way. We now seek such explanations.

One possible explanation for the magnitude of predicted total pressure dependent outflows is the influence of so-called ‘pseudofacility’. Pseudofacility is defined as the ‘excess facility’ of outflow in a measurement of outflow facility due to the reduction in net fluid production as a result of IOP elevation [31]. Based on earlier work by Barany (e.g. [92]), Brubaker (1970) helpfully explains how to estimate ‘true outflow facility’, ‘pseudofacility’ and ‘total outflow facility’, and then makes the necessary experimental measurements on rhesus monkeys, estimating total outflow facility at 0.67 microlitres/min/mm Hg and estimating pseudofacility at 0.12 microlitres/min/mm Hg i.e. about 18% of the total outflow facility [25]. If the same fraction of pseudofacility occurred in human eyes, then about 0.18 times 6.6 = 1.2 microlitres/min could be explained by pressure dependent flow through the ciliary body epithelium.

Says Brubaker (1970): ‘the total outflow facility can be measured by comparing two steady states at different intraocular pressure [with constant EVP], while true outflow facility can be measured by comparing two steady states at different episcleral venous pressures [with constant IOP]. The pseudofacility is the difference between the two measurements.’ Providing one recognises that true outflow facility includes flow through to the choroid as well as through the anterior pathways, and providing membrane reference pressures for all pathways are controlled by the neck band and mirror episcleral venous pressure, and provided the flow is across a simple ideal membrane, this is a logical enough way of deducing the pressure dependent flow through the ciliary epithelium.

But all outflow pathways from the eye are complicated—one pathway involves two non-ideal membranes with an interstitial fluid with protein between (e.g. retinal pigmented epithelium route), one pathway involving more than two membranes (e.g. ciliary body), and some pathways remain uncertain as to exactly what is going on despite intensive research over many decades (i.e. uveoscleral outflow and trabecular meshwork outflow for humans). So despite the certainty with which these experimental methods are specified to estimate ‘total outflow facility’ and ‘pseudofacility’ by Barany (1963) and Brubaker (1970), the exact relationship between these test measurements and what this tells us about fluid outflow from the *in vivo* eye is somewhat uncertain. Indeed, when developing his analysis Barany makes remarks to the effect that his proposed conceptual model is ‘only a first approximation and undoubtedly will have to be refined’ [92]. It is now known the complexity of fluid transfer from the eye was not fully appreciated in the 1960s. For example ion pumping across the retinal pigmented epithelium was not known until 1979 [93], while aquaporins were not discovered until 1992 by Peter Agre [94].

We already know that the Na^+^K^+^ATPase ion pumps are not influenced by physiological hydrostatic pressures experienced by the eye. Net fluid production may decrease either due to an increase in driving pressure from the eye across the non-pigmented epithelium at the ciliary body, or because net fluid production may decrease due to inadequate availability of ATP to maintain aerobic metabolism. The most likely cause of inadequate ATP is a reduction in blood flow, leading to a decrease in the local partial pressure of oxygen and local hypoxia at the ciliary body. On this topic, Kiel et al (2010) reports Moses as stating that:

…the production of aqueous humor formation decreases slightly as intraocular pressure increases until the region of ciliary blood pressure is approached’…[while Kiel later states]…that recent studies of the relationship between ciliary blood flow and aqueous humor production support the prediction of Moses’ iconic graph.

Moses’ ‘iconic graph’ (Fig 1, Kiel et al (2011)) shows the aqueous humor production decreasing slightly (by about 0.01 microlitres/min/mm Hg; at 15 mm Hg this is 0.15 microlitres/min, or about 0.15/2.6 = 6% of reported aqueous production (and only about 2.3% of our model predicted pressure dependent outflow), considerably less than that the 18% reported by [25]. This same figure also shows aqueous humor production dropping precipitously above about 50 mm Hg as the ciliary blood flow is interrupted.

Pseudofacility may be estimated from ‘first-principles’, providing the osmotic suction is known and some additional assumptions are made. As the osmotic suction is not known, to obtain an approximate first estimate we assume that the osmolality difference across the ciliary epithelium is 5 mOsm (i.e. the same as found at the choroid plexus epithelium in the ventricles of the brain [52]) and that this creates an osmotic suction of about 100 mm Hg across the ciliary body, drawing water into the eye. Because the hydraulic conductivity of ciliary body (and indeed all typical membrane) is the same in both directions, the ratio of the pressure dependent outflow across the ciliary body (equal to *f* times total inflow), is given by $C_{cb}^{SL}(p-p_{ref-cb})/(fC_{cb}^{SL}(100-(p-p_{ref-cb}))$, where *f* is the fraction of total ciliary body fluid production that exits the eye by pressure dependent outflow pathways.

Assuming *f* is 0.7 (which can be checked by calculations below), IOP is 15 mm Hg and the interstitial reference pressure near the inner surface of the ciliary epithelium is 4 mm Hg (0/8 mm Hg, where 8 mm Hg is close to the hydraulic conductivity weighted average reference pressure for the whole (human) eye at IOP of 15 mm Hg), then outflow at the ciliary body is estimated to be about 18% (25/11%) of the total pressure dependent outflow, about the same as the pseudofacility experimentally estimated by Brubaker (1970). Assuming *f* is 1.0, IOP is 15 mm Hg and the interstitial reference pressure near the inner surface of the ciliary epithelium is 4 mm Hg (0/8 mm Hg), then outflow at the ciliary body is estimated to be about 12% (18/9%) of the total pressure dependent outflow Though there are a number of assumptions in this first-principles analysis it appears plausible, so in the following analysis we therefore lean towards Brubaker’s (1970) estimate for pseudofacility rather than some much lower estimates, and settle on 12.5% as a reasonable estimate of the total outflow facility being pseudofacility. Given pseudofacility explains about 12.5% of estimated pressure dependent outflow (for 6.6 microlitres/min) that is 0.83 microlitre/min. But clearly pseudofacility cannot by itself explain the discrepancy, so we seek alternative explanations.

Given that fluorometric estimation of aqueous outflow through the anterior chamber of the eye is the basis for estimation of aqueous inflow [64, 95], another possible explanation is that the measured aqueous outflow through the anterior chamber does not reflect the total production of fluid by the ciliary body. This concept would suggest that a considerable fraction of fluid produced by the ciliary body travels posteriorly through the vitreous humor, exiting through the retinal-choroidal route. This seems plausible, as there are a large number of papers supporting the notion of a posterior flow through the vitreous towards the retinal surfaces [10, 18, 96–99]. In addition, there are also many papers reporting measurements of flow across the pigmented retinal epithelium that suggest both pressure dependent and pressure independent flows occur from the vitreous through the retina, across the pigmented retinal epithelium and Bruch’s membrane, into the interstitial choroidal fluid and finally into the choriocapillaris [8, 9, 12–18, 93, 100–103].

For example Tsuboi (1987) measured both the pressure dependent and independent flows across retinal pigmented epithelium for adult dogs weighing 15 Kgs to 30 Kgs. The pressure independent flow from retina to choroid is reported to be 0.11 microlitres/min/cm^2^, and the hydraulic filtration coefficient for the retinal pigmented epithelium into the choroid (*L*_*p*−*RPE*_) is reported to be 0.0126 microliters/min/cm^2^/mm Hg. For cynomolgus monkeys, Emi et al (1989) reports that at an intraocular pressure of 15 mm Hg, the driving pressure (*p* − *p*_*ref*_) from vitreous to choroid is about 5 mm Hg. Combining these estimates with the surface area of the retina in the human eye (reasonably estimated at 12 cm^2^ [104], but could range between 10 and 14 cm^2^ depending on eye size), this leads to an estimated pressure independent flow in the human eye of around 1.3 microlitres/min, and a pressure dependent flow from the vitreous into the choroid to be around 0.8 microlitres/min, giving at total flow through the posterior route of about 2.1 microlitres/min. However Chihara et al (1985) reports resorption of sub-retinal fluid for *in vivo* human eyes occurs at the rate of 0.18 microlitres/min/cm^2^ (about half the volume of the vitreous is replaced each day [18], n = 10) leading to a directly estimated total outflow through the retinal pigmented epithelium for the human eye of around 2.5 microlitres/min, which is similar though somewhat higher than the previous estimate of the total outflow for humans based extrapolated from measurements made on dog tissue.

We mention here that it is actually difficult to classify outflows as pressure independent and pressure dependent at the retinal pigmented epithelium, because if salt is not pumped across the retinal epithelium while water flows through the membrane, salt accumulates on the vitreous side of the membrane and the vitreous and retinal tissues will experience hypertonicity. It is possible that the local hypertonicity at the membrane influences ion pump density and so the pumping rate at the retinal pigmented epithelium, as suggested by Na-K-ATPase mRNA upregulation for dog lens epithelial cells exposed to salt hypertonicity [105], but whether this happens at the retinal pigmented epithelium is currently unknown [57]. In other words, it is difficult to classify outflow as pressure dependent or independent without knowing the relative importance of all the drivers for all ion transporter densities, and so the drivers for pumping rates by the epithelial cell sheet [106]. In this context, if we regard outflow through the retinal pigmented epithelium as entirely pressure independent, then *f* can be estimated to be about 6.6/(6.6+2.5) = 0.7, but if this outflow is regarded as entirely pressure dependent then *f* is about 1.0 (see pseudofacility analysis in this section for definition of *f*).

If the outflow through the retinal pigmented epithelium is regarded as pressure dependent and equal to about 2.5 microlitres/min, then to a first approximation, this suggests that approximately equal amounts of fluid produced by the ciliary body exit through the anterior and vitreous chambers of the eye. But an estimate of 2.5 microlitres/min of outflow through the retinal pigmented epithelium *in vivo* still explains only a fraction of the discrepancy in pressure dependent flow noted above. Assuming a pressure independent flow anteriorly through the uveoscleral route of 1 microlitre/min [107] is in fact pressure dependent (see Discussion above), and that the estimated 2.5 microlitres/min outflow through the retinal pigmented epithelium is pressure dependent, this means most of the estimated pressure dependent outflow has been accounted for—that is, 2.5 (anterior routes) + 2.5 (retinal pigmented epithelium) + 0.83 (possible pseudofacility) giving a total of 5.83 microlitres/min. The estimated *in vivo* outflow of 5.83 microlitres/min accounts for (5.83/6.6) about 90% of the estimated pressure dependent flow. On the other hand, if unconventional outflow and outflow across the retinal pigmented epithelium are regarded as pressure independent, then only (1.6+0.8+0.83)/6.6) about 50% of the estimated pressure dependent outflow is explained.

Clearly the above observations on basic eye physiology appear to have gone some way to explaining the total pressure dependent outflow estimated here at 6.6 microlitres/min (and does appear to explain the median estimate of pressure dependent outflow of 5.6 microlitres/min). But however outflow is classified (pressure dependent or pressure independent), our analysis of the pressure dependent outflows may not entirely explain the estimated pressure dependent outflow (and if some outflow is regarded as pressure independent, the discrepancy between reported aqueous production and predicted total outflow has only widened). What else might provide an explanation? Could there be an additional source of fluid in the eye, which is possibly of greater magnitude in aged people? In this context, it appears reasonable to postulate that the total fluid formation rate within the eye is the sum of ciliary body production and leakage of fluid from the retinal vasculature.

In the normal eye, the blood-retinal barrier is intact and fluid leakage is negligible, however in aged eyes, it is known that various pathologies may arise that entail fluid leakage from the retinal vasculature. Crucially, knowing the physiology of transport across membranes involves aquaporins, it a small step to then expect that increases in vascular and epithelial permeability will be accompanied by increases in aquaporin density (e.g. AP1/AP4 density) [14, 108–110]. Indeed this appears to be the case (e.g. [111, 112]). It is known that VEGF and some inflammatory mediators increase the expression of aquaporins in endothelial and epithelial cells [16, 113–116]. Developing sub-clinical pathologic states would allow increasing pressure dependent fluid leakage from the retinal vasculature into the retina (providing another fluid source within the orbit [16, 115, 116]) while increasing the hydraulic filtration coefficient for the pigmented retinal epithelium (increasing the pressure dependent outflow facility of the posterior chamber of the eye) [16, 117].

It therefore appears reasonable to suppose that in aged eyes the net fluid production within an eye is the sum of ciliary body production (which for normal eyes is the sum of normal pressure dependent and pressure independent outflows) and vascular leakage, and for aged eyes with normal IOP this increased rate of fluid production due to vascular leakage is balanced by an increase in pressure dependent outflow across the retinal pigmented epithelium. If we add this incremental fluid transport to the normal fluid movement across the retinal pigmented epithelium due to increasing $C_{ch}^{SL}$, it appears plausible that the total pressure dependent outflow in apparently normal aged eyes could reasonably be expected to reach 6.6 microlitres/min, and possibly more.

## Conclusions

In this paper we have developed a new theory of pressure dependent outflow based on the difference in the differential rates of change of IOP and the membrane reference pressure with respect to changing IOP. Though an alternate functional relationship may be required in some circumstances, as a good first approximation we have fitted a simple exponential decay function to approximate the local outflow facility of the eye at pressure *p*, denoted *C*^*p*^. Averaging local outflow facilities across a specified pressure range defines the average outflow facility, denoted ${\bar{C}}_{p_{1}}^{p_{2}}$. For our model, we find there are three key parameters controlling pressure dependent outflow: (i) the hydraulic conductance for the whole eye, denoted $C_{T}^{SL}$ (microliters/min/mm Hg), (ii) the exponential decay constant, denoted *α* (mm Hg)^-1^ and (iii), and no flow reference intraocular pressure denoted *p*_*T*_ (mm Hg). We then investigated the parameters controlling pressure dependent outflow, and fit model parameters to published data. We have employed animal and human *in vivo* data to make first estimates of all three key parameters $C_{T}^{SL}$, *α* and *p*_*T*_. Using a mixture of both animal and human data our first-principles estimate for *p*_*T*_ is 3 mm Hg, and *α* is found a range of 0.04 to 0.13, with a mean estimate of 0.07. A reanalysis of Barany (1964) *in vivo* data for Vervet monkeys suggests *α* is about 0.05, and $C_{T}^{SL}$ about 1.0. A reanalysis of Brubaker’s (1975) data on enucleated eyes suggests *α* is about 0.04, $C_{T}^{SL}$ about 0.70, when *p*_*T*_ is assumed to be 3 mm Hg. Finally, based on the *in vivo* human eye data presented in Dastiridou et al (2013) and Karyotakis et al (2015), our analysis suggests that for a group of cataract patients, average age over 70 years and with no known eye disease, *α* is about 0.07 (0.05 and 0.075), while mean $C_{T}^{SL}$ ranges between 1.0–1.7 (median $C_{T}^{SL}$ is probably around 0.85) (see summary of parameter estimates Table 2). If *α* and $C_{T}^{SL}$ both increase with age, a more rapid increase in *α* relative to $C_{T}^{SL}$ may lead to unstable IOPs and ocular hypertension. We have employed the estimates of $C_{T}^{SL}$, *α* and *p*_*T*_ to calculate average outflow facilities for the *in vivo* eye, together with estimating the total pressure dependent outflow. While acknowledging limitations of these estimates, using this approach we have found the pressure dependent outflows are around twice as large as fluorometric estimates for aqueous outflow. We have sought possible explanations for this discrepancy and concluded that it appears likely that the discrepancy may be explained by a combination of normal pseudofacility, normal fluid movement through the vitreous exiting the eye via the retinal pigmented epithelium, and increasing retinal pigmented epithelial flows as a result of age related disease processes. We hope further theoretical and experimental data can help refine and extend the theory presented here (e.g. to include another model parameter *p*_*B*_), and in doing so provide a sound theoretical framework for a fuller understanding of fluid dynamics for the eye. While much remains to be done to understand the variety of physiological mechanisms potentially explaining model parameters, with further research the theoretical framework described herein may enable more accurate estimation of pressure dependent outflows for human eyes that aids diagnosis, monitoring and prognosis of glaucoma.

## Supporting information

S1 Supporting Information Ocular Rigidity (DOCX)
