# Supplementary material for: Estimating outflow facility through pressure dependent pathways of the human eye
Source: PLoS One. 2017 Dec 20;12(12):e0188769. doi: 10.1371/journal.pone.0188769 (PMC5738051; doi:10.1371/journal.pone.0188769)
Supplement: S1 Supporting Information Ocular Rigidity — (DOCX) [file pone.0188769.s001.docx]

## S1 Supporting information

## Pressure dependent ocular rigidity for the *in vivo* human eye

Here we develop the theory of pressure dependent ocular rigidity following the approach of Silver and Geyer (2000). Consider the flow of an incompressible fluid through the eye. The mass balance equation of the fluid states that the rate of change of volume of the eye is equal to the difference in the rates of fluid volumes entering and leaving the eye, viz,

where is the rate of change of eye volume, and and are the rates of fluid volumes entering and leaving the eye. The change of eye volume associated with a change in pressure can be included using the chain rule, viz,

where $\frac{dV_{e}}{dp}=C_{e}$ is the ocular compliance at pressure $p$ (microliters/mm Hg). We now seek a suitable constitutive relationship between eye volume and pressure. Friedenwald (1937) suggested that the volume strain of the eye is proportional to the common logarithm of the pressure. That is,

$$\varepsilon_{v}=\frac{\Delta V_{e}}{V_{e-ref}}=\frac{1}{\hat{K}}\ln\frac{p}{p_{e-ref}}=\frac{2.3}{\hat{K}}\log\frac{p}{p_{e-ref}}$$

where $\varepsilon_{v}$ is the volume strain of the eye, $p_{e-ref}$ is the unit reference pressure in mm Hg, $V_{e-ref}$ is the reference volume at the unit reference pressure. Note the constant $\hat{K}$ is without units. Rearranging leads to:

$$\Delta V_{e}=\frac{V_{e-ref}}{\hat{K}}\ln\frac{p}{p_{e-ref}}=\frac{1}{K}\ln\frac{p}{p_{e-ref}}=\frac{2.3}{K}\log\frac{p}{p_{e-ref}}$$

where $K=\hat{K}/V_{e-ref}$ is known as the ocular rigidity (with units of per microliter). While practical, the ocular rigidity defined in this way unfortunately mixes a ‘material parameter’ $\hat{K}$ with the eye volume $V_{e-ref}$. We note that the so-called ‘Friedenwald coefficient’ is $K/2.3$.

Differentiation of equation leads to,

$$\frac{d\Delta V_{e}}{dp}=\frac{V_{e-ref}}{\hat{K}p}=\frac{1}{Kp}$$

The eye volume can be estimated from an ellipsoid as $V_{e-ref}=(\pi/6)abc$, where $a$,$b$ and $c$ are the major, intermediate and minor axes of the ellipsoid. A suitable reference pressure is 15 mm Hg which is the normal *in vivo* IOP for the human eye. Friedenwald (1937) had recognised that the eye is rather stiff, so that the fractional change in IOP is much greater than the fractional change in volume. In this case, the eye volume may be approximated as constant. However later experimental measurements of the volume strain *in vivo* demonstrated that the ocular rigidity *in vivo* is in fact a function of pressure. Focusing on the ‘material parameter’ $\hat{K}$, Silver and Geyer fitted a straight-line relationship to the 121 data points obtained from 21 living eyes published in the available literature (average age 56, age range 29 to 82 years). (Silver and Geyer 2000) proposed:

$$\frac{2.3}{\hat{K}}=\frac{2.3}{KV_{ref}}=(1.12\times10^{-2}+8.99\times10^{-5}p)$$

Substituting equation for the ocular rigidity into equation describing the change in eye volume with pressure, and then integrating (assuming a reference volume of 15 mm Hg) immediately leads to,

$$\Delta V_{e}=V_{e-ref-15mmHg}(c_{-1}^{*}+c_{0}^{*}\ln p+c_{1}^{*}p)$$

where $c_{-1}^{*}=-8.03\times10^{-3}$ and $c_{0}^{*}=4.87\times10^{-3}$ and are unitless constants, and the constant $c_{1}^{*}=3.90\times10^{-5}$ has units of mm Hg^-1^. Employing a reference eye volume ($\bar{V}_{e-ref-15mmHg}$) as the average across both sexes at 15 mm Hg (i.e. 6200 microlitres; mean volumes 6500 microlitres for men and 5900 microlitres for women (Silver and Geyer 2000)), leads directly to the equation for estimated average volume change of the eye with pressure, viz,

$$\Delta V_{e}=c_{-1}+c_{0}\ln p+c_{1}p=-85.17+30.2\ln p+0.242p$$

where $c_{-1}=-85.17$ and $c_{0}=30.2$ have units of microliters, and $c_{1}=0.242$ has units of microliters/mm Hg. We may now again differentiate this equation to estimate the revised average ocular stiffness for an averaged size eye as:

$$\frac{dV_{e}}{d\ln p}=p\frac{dV_{e}}{dp}=\frac{1}{K}=c_{0}+c_{1}p=30.2+0.242p$$

where $1/K$ and has units of microliters. We mention that this equation means that for the ocular rigidity is to be constant (i.e. $c_{1}=0$, as most often assumed for standard constitutive pressure-volume relationships), the fractional decrease in $dV_{e}/dp$ must exactly match the fractional increase in IOP. And we also note that these average rigidity parameters mean the average ocular rigidity decreases with increasing IOP (e.g. at 15 mm Hg the ocular rigidity is 0.0296, while at 40 mm Hg it is 0.0251). Upon rearranging the previous equation we have,

$$\frac{dV_{e}}{dp}=\frac{1}{pK}=C_{e}=\frac{c_{0}}{p}+c_{1}=\frac{30.2}{p}+0.242$$

where $C_{e}$ is an ocular compliance that now has units of microliters per mm Hg, which can be employed in equation .

We also notice in passing that if $c_{0}=0$ in equation , then the volume and pressure change are linearly related to each other by $C_{e}$. As mentioned above, equation means that ocular compliance increases—and the stiffness decreases—as the intraocular pressure increases. The revised equation developed by Silver and Geyer (2000) estimates the *in vivo* ocular rigidity $K$ at 15 mm Hg to be around 0.0296 per microlitre, substantially smaller than the original estimates by Friedenwald (1937) based on *ex vivo* tests (ocular rigidity is 0.021 (Friedenwald coefficient) times 2.3 = 0.0483 (rigidity coefficient for natural logarithms) i.e. $c_{0}=20.70$ and $c_{1}=0$)). So for example, the change in eye volume between 15 mm Hg and 40 mm Hg is 35.67 microlitres for Silver and Geyer (2000), and 20.30 microlitres using the Friedenwald (1937) rigidity, as the ‘Friedenwald eye’ is much stiffer than the *in vivo* eye (see Fig 2 Silver and Geyer (2000)). This is because Friedenwald tested enucleated eyes, while Silver and Geyer’s data was obtained from tests on *in vivo* eyes, which are substantially more compliant, probably because blood is progressively squeezed from the uveal vasculature with increasing IOP. We also mention that an apparently small change in $c_{1}$ from plus 0.242 to minus 0.242 (while $c_{0}$ remains unchanged) brings the Silver and Geyer pressure-volume model (eye volume change between 15 mm Hg and 40 mm Hg is then 23.57 microlitres), close to the Friedenwald pressure volume model (eye volume change between 15 mm Hg and 40 mm Hg remains 20.30 microlitres). So clearly $c_{1}$ is an important parameter. It is known that accurate estimation of ocular stiffness plays an important role in the accurate estimation of outflow facility.

We mention that fitting the Silver and Geyer (2000) model to pressure-volume data can result in more than one parameter solution set. For example, the pressure-volume data shown in figure 1 of Detorakis et al (2013) can be reasonably approximated with $c_{0}=70$ and $c_{1}=-1.05$. In this case the ocular rigidity increases with increasing pressure (e.g. at 15 mm Hg ocular rigidity is 0.0184, and at 40 mm Hg it is 0.0357). But the same data can also be reasonably well-fitted with $c_{0}=45$ and $c_{1}=0.2$. In this case, the ocular rigidity decreases with increasing pressure (e.g. at 15 mm Hg ocular rigidity is 0.0203, and at 40 mm Hg is 0.0189). This mathematical non-uniqueness can be resolved by choosing the most appropriate physiological solution, which is usually the solution with decreasing ocular rigidity with increasing IOP.

Finally we mention that apart from proposing a new constitutive pressure-volume relationship for the eye, Silver and Geyer (2000) separated the influence of volume from the ‘material property’ of responsible for ocular rigidity. Because the average volume of women’s eyes (i.e. 5900 microlitres) is significantly less than men’s eyes (i.e. 6500 microlitres), assuming exactly the same ‘material properties’ for the eye (including drainage of uveal blood with increasing IOP), the ocular stiffness of women’s eyes is significantly greater than the stiffness of men’s eyes. So for example using Silver and Geyer (2000) estimates, the change in volume of average women’s eye due to a change in pressure from 15 mm Hg to 40 mm Hg is 33.90 microlitres, while for men it is 37.44 microlitres (about a 10% decrease in eye stiffness for men relative to women). This is purely a geometric effect of greater eye curvature in women’s eyes relative to men’s eyes, which decreases both uveal surface area and scleral wall tension (for the same IOP).
